# Mixed methods process theory evaluation to explore the implementation issues of the Needs Assessment Tool-Cancer (NAT-C) in primary care for people with cancer

**DOI:** 10.1136/bmjopen-2025-113686

**Published:** 2026-04-08

**Authors:** Flavia Swan, Joseph Clark, John Blenkinsopp, Amanda J Farrin, Alexandra Wright-Hughes, Emma McNaught, Terry McCormack, Miriam J Johnson

**Affiliations:** 1 Wolfson Palliative Care Research Centre, University of Hull, Hull, UK; 2 Oslo New University College, Oslo, Norway; 3 Edinburgh Clinical Trials Unit, University of Edinburgh Division of Clinical and Surgical Sciences, Edinburgh, UK; 4 Clinical Trials Research Unit, University of Leeds, Leeds, UK; 5 Hull York Medical School, University of Hull, Hull, UK

**Keywords:** Cancer, Primary Care, PALLIATIVE CARE

## Abstract

**Background:**

The Needs Assessment Tool-Cancer (NAT-C) is a consultation guide to identify, triage and reduce unmet patient needs.

**Objectives:**

We aimed to assess NAT-C fidelity, mechanisms of action and implementation issues in UK primary care as part of a clinical and cost-effectiveness cluster randomised controlled trial of the NAT-C for people with cancer compared with usual care (registration: ISRCTN15497400).

**Methods:**

Design: a mixed-methods process evaluation informed by normalisation process theory (NPT). Setting: 21 participating general practices in England were randomised to be trained to conduct an NAT-C guided consultation with people with cancer (excluding those in remission). General practitioner fidelity of intervention and clinical action resulting from the NAT-C consultation was noted. Two Normalisation MeAsure Development Questionnaire surveys were distributed to trained clinicians before (Survey 1) and after delivery of ≥2 NAT-C consultations (Survey 2). Semi-structured interviews were conducted with clinicians (post delivery ≥2 NAT-C consultations) and key stakeholders in primary and cancer care. Fidelity, action and paired before/after survey data were analysed using descriptive statistics. Interview data were analysed using a deductive thematic framework approach (NPT-informed). Data were narratively synthesised with cross-tabulated key findings.

**Results:**

Of the 360/376 (96%) NAT-C consultations delivered, 258/360 (72%) resulted in clinical action, including 50 (13%) external referrals. 14 paired before (Survey 1, n=53) and after (Survey 2, n=29) responses. Survey 1 showed positive responses across all NPT domains, but while continuing to see relevance, usefulness and legitimacy, Survey 2 highlighted concerns about insufficient resources and management support. 16 clinician participants (eight GPs, eight key stakeholders; 50% male) completed interviews. Following synthesis, we identified five themes: (1) the perceived value of the NAT-C; (2) ‘champions’ are important at all levels (practice, regionally and nationally); (3) research evidence is seen as important, but influences implementation indirectly through policy, clinical guidelines and resourced initiatives; (4) adequate resources are fundamental for implementation beyond practice level and (5) NAT-C practicalities; training is adequate, but robust functional information technology systems are needed.

**Conclusion:**

Implementation requires champions and clinicians ‘buy-in’ to the patient value to legitimise use. In the context of current primary care pressures, resources were seen as essential to embed the NAT-C, but financial incentives were viewed with mixed feelings.

**Trial registration number:**

ISRCTN15497400.

**STRENGTHS AND LIMITATIONS OF THIS STUDY**

- The mixed-methods synthesis is underpinned by normalisation process theory, adding value to the understanding of potential implementation issues.
- Paired surveys and interviews throughout the course of the study allow the impact of changing context to be seen.
- The wider stakeholder interviews provide rich data and depth of insight into the national and regional contexts.
- Recruitment difficulties meant that the target interview sample was not reached.
- The viewpoint of other clinicians, such as practice nurses, is unknown as none were recruited.

## Background

 Many people with cancer have unmet needs, most commonly in the recently diagnosed or with advanced disease.^1^ Unmet needs are likely to increase in number and complexity as cancer prevalence increases (estimated 35.3 million by 2050 globally^2^), ageing populations with multiple comorbidities and the long-term consequences of treated cancer.

In the UK and other jurisdictions with universal primary care health coverage, primary care is ideally positioned to address unmet needs.^3^ Cancer care reviews (conversations between newly diagnosed cancer patients and primary care clinicians, attracting Quality Outcomes Framework (QOF) payments) have been widely adopted in UK primary care. However, there is insufficient evidence of benefit^4^ and variation in implementation fidelity.^4^

The challenges of implementing complex interventions in primary care are well documented. An overview of systematic reviews of implementing clinical practice guidelines into primary care practice showed many barriers, including poor intervention real-world applicability, lack of knowledge, skills and motivation, poor adherence, and inadequate funding.^5^

The Needs Assessment Tool-Cancer (NAT-C) is a clinical consultation guide adapted and validated for UK primary care.^6^ Following feasibility testing,^7^ we undertook a multisite cluster randomised controlled trial (cRCT) (CANAssess2) testing the effectiveness and cost-effectiveness of the NAT-C in reducing unmet patient and carer need compared with usual care. The trial found no benefit for the primary endpoint of 3 months, but patient-relevant benefits in the severity of unmet need, symptom management and quality of life at 6 months were seen.^8^ Although quality of life improvements were small, the health economic evaluation showed a high probability of cost-effectiveness at National Institute for Clinical Effectiveness (NICE) thresholds.^8^ We undertook a process evaluation to explore (1) the adequacy of NAT-C training, (2) intervention fidelity and (3) possible mechanisms of action and implementation issues.

## Methods

We conducted a mixed-methods process evaluation of general practitioner (GP) practices (family practices) allocated to the NAT-C intervention in the CANAssess2 trial.^8 9^ The trial is reported elsewhere.^8^ Normalisation process theory (NPT)^10^ informed data collection and analysis.

### Study design and methods

#### NAT-C intervention, training and fidelity

The NAT-C is a one-page tool for assessment of patients’ and carers’ holistic needs.^6 8^ It triages between needs manageable within the primary care team and those requiring external referral, for example, palliative care, psychology and benefits advice. Resulting clinical action was agreed between clinician and patient and documented in the completed NAT-C (using electronic medical record (EMR) template, or paper version uploaded to the EMR).

21 intervention-allocated practices across two large regions in northern England nominated at least one clinician for live NAT-C training (maximum 1 hour) facilitated by a researcher (JC, TM). A PowerPoint presentation provided information for NAT-C delivery and included a video of a real-life NAT-C-guided clinical consultation. A prerecorded narrated version of the NAT-C training was sent to additional clinicians nominated but unable to attend live training.

All practices were provided with NAT-C resources (online supplemental file 1). Evaluation of intervention delivery, uptake and fidelity included collection and reporting of data on the number of NAT-C trained clinicians in each practice, completed NAT-C consultations, length of NAT-C consultations, and referral patterns and actions following the consultation. Information was collected on the clinician role, training method and the number of days between training and the first NAT-C consultation.

#### Surveys 1 and 2

A survey based on the Normalisation MeAsure Development Questionnaire (NoMAD) tool^11^ was developed during feasibility testing,^9^ available online using the Qualtrics platform.^12^ Clinicians were asked their level of agreement with statements about the NAT-C, NAT-C training and implementation. Free-text responses were permitted.

All clinicians who completed NAT-C training were invited to complete the survey following completion of training (Survey 1) and after ≥2 NAT-C consultations (Survey 2), forming convenience samples. Completion was understood as implied consent.

Permission to send Survey 2 and the interview information sheet/invitation was sought at the end of Survey 1. Survey 1 opened in October 2020 (first practice trained) and closed in March 2023 (last practice trained). Survey 2 was open from September to November 2023. All survey data were anonymised.

#### Interviews

Eligible clinicians were from trial GP sites, had received NAT-C training and had conducted ≥2 NAT-C guided consultations. Planned purposive sampling of clinicians (by doctor and nurse) was not possible due to a lack of response from nurses. Eligible key stakeholder participants were identified through known contacts or information available in the public domain and were emailed an invitation with a study information sheet. Purposive sampling of key stakeholders aimed to include those from local commissioning groups, general practice federations, the National Cancer Research Institute’s primary care group, the Royal College of GPs and Macmillan primary care leads.

Semi-structured, online interviews with consenting (verbally recorded prior to the interview) participants were conducted by two researchers, JC (research fellow, social science background, male) and FS (research fellow, physiotherapy clinical background, female), with previous interviewing experience, using topic guides developed from the literature and team expertise, covering the NPT constructs (online supplemental file 2).

Clinician interviewees were asked about their views on the adequacy of NAT-C training, how they used the NAT-C and their views on potential implementation of the NAT-C into routine primary care. Stakeholder interviews focused on structural and policy issues relevant to the potential implementation of the NAT-C in GP practices nationwide.

All interviews were audio-recorded and transcribed (anonymised) using Microsoft (MS) Teams.

#### Data analysis

##### Quantitative data

We used descriptive statistics (numbers, percentages, mean and SD, median and quartiles) to present training, intervention fidelity and survey data.

##### Interviews

Interview data and survey open-ended text responses were analysed together using codebook thematic analysis^13 14^ informed by NPT constructs (online supplemental file 3).^15^

Data were managed using NVivo V.14,^16^ and analysis involved the following steps: transcription and anonymisation, familiarisation, line-by-line coding, developing an analytical framework, indexing, charting and interpretation. Coding was mapped to NPT constructs and themes developed. FS and MJ coded four transcripts independently and agreed on a code list. FS, JC, MJ and JB had ongoing discussions regarding coding reliability and construct mapping to ensure transparency.

##### Data synthesis

The training, fidelity, survey and interview data were cross-tabulated and summarised.

### Patient and public involvement

An experienced lay representative was part of our funding application. She has reviewed and edited public-facing study documentation and attended our Trial Management Group meetings, with public patient involvement as a standing item. A further lay representative took part in our Trial Steering Committee.

### Ethical approvals and trial registration

The trial received ethical approval from the London-Surrey National Health Service (NHS) Research Ethics Committee (20/LO/0312). The trial was conducted in accordance with the principles of Good Clinical Practice and the Declaration of Helsinki and was registered on 07 April 2020 (ISRCTN15497400).

The process evaluation study is reported according to the Standards for Reporting Implementation Studies (StaRI) guidelines.^17^

## Results

From 21 practices, 53 clinicians were trained to deliver a NAT-C consultation. Most completing training were GPs, 35 (66%) and practice nurses, 13 (24.5%) (table 1). All training sessions were facilitated live, online (MS Teams) due to COVID-19 constraints (apart from one person who completed pre-recorded training).

**Table 1:** Intervention clinician training

Total practices (n=21)	
Total clinicians trained	53
Per practice mean* (SD)	2.5 (1.36)
Median* (range)	2.0 (1.0–6.0)
IQR	(2.0–3.0)
Missing	0
Clinician roles (n=53)	
GP	35 (66%)
Practice nurse	13 (24.5%)
Advanced nurse practitioner	4 (7.5%)
Physician associate	1 (2%)
Of those trained† (total trained (n=53))	
Live facilitated online	52 (99%)
Prerecorded	1 (1%)
Days between training and first NAT-C appointment (n=32)	
Mean (SD)	105.7 (47.93)
Median (range)	97.0 (46.0–230.0)
IQR	(67.0–134.0)
Missing	22‡

* Per practice.

† There were no refresher training sessions, although they could have been delivered on request.

‡ Reasons for missing: Clinician completed training but conducted no appointments. There were some NAT-Cs where the name was unclear (ie, two doctors with the same surname).

The number of days between training and first NAT-C appointment was mean (SD) 105.7 (47.93), median (range) 97.0 (46.0–230.0).

The NAT-C was delivered to 360/376 (96%) participants in intervention practices (n=16 withdrawal/did not attend), taking a median 24 min (IQR 20–30) and resulting in external referrals for 50/360 (14%). Most referrals, where specified, were to a specialist palliative care service (n=10) or psychologist (n=7). Action was taken for 258/360 (72%) participants, either direct management (232/360 (64%)) or by another team member (61/360 (17%)) (online supplemental file 4, tables 1, 3 and 4).

The NAT-C was performed at least once by 32/53 (60%) of the trained clinicians. The number of NAT-Cs performed by clinicians was mean (SD) 11.25 (8.95), median (min–max) 9 (1–32), IQR 4–16.5. See online supplemental file 4, table 2.

Patient-participant characteristics from practices randomised to use the NAT-C are described elsewhere^8^ but are summarised here: 194/376 (52%) participants (mean age 66.6 years; males 177/376 (47%); white ethnicity 371/375 (99%); advanced/metastatic disease 165/366 (52%)) had at least one moderate–severe unmet need at baseline.

### NoMAD surveys 1 and 2

**Figure 1:**
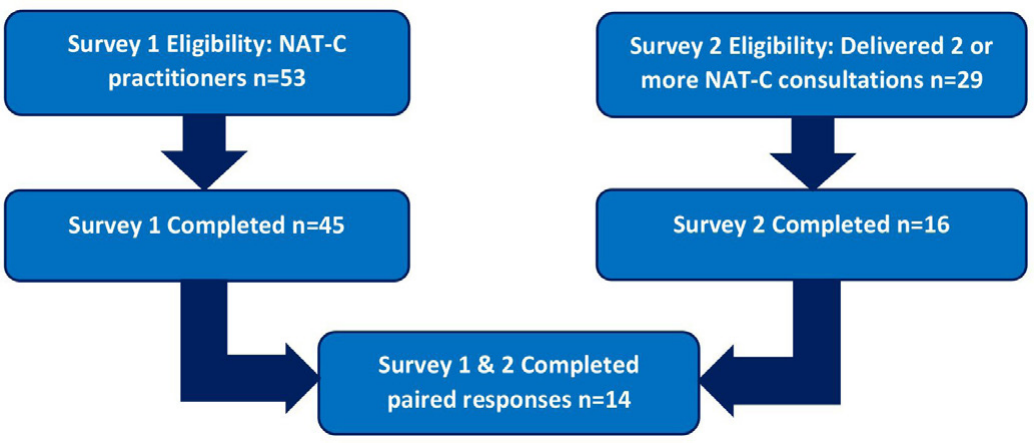
Flow diagram of surveys 1 and 2 participant eligibility and completion. NAT-C, Needs Assessment Tool-Cancer.

Of those eligible, 45/53 (84.9%) NAT-C trained clinicians completed Survey 1, and 16/29 (55.2%) completed Survey 2 to give 14 paired responses (figure 1). All were completed online. There were two missing responses for one question in Survey 1 only.

Most survey participants were GPs and nurses with a wide range of primary care experience (see table 2). Those completing both surveys were mainly GPs (85.7%), reflecting the professional group conducting most NAT-C consultations.

**Table 2:** Normalisation MeAsure Development Questionnaire surveys 1 and 2 clinician participant characteristics

Clinician participant characteristics N (%)		Survey 1 (n=45)	Survey 2 (n=16)	Surveys 1 and 2 paired ‘before/after’ responses (n=14)
Main role	General practitioner	31 (68.89)	13 (81.25)	12 (85.7)
	Practice nurse	11 (24.44)	2 (12.50)	2 (14.3)
	Other	3 (6.67)	1 (6.25)	–
Length of time working in general practice	Less than 1 year	8 (17.78)	0 (0.00)	3 (21.45)
	1–2 years	12 (26.67)	1 (6.25)	1 (7.1)
	3–5 years	4 (8.89)	5 (31.25)	3 (21.45)
	6–10 years	8 (17.78)	7 (43.75)	5 (35.7)
	11–15 years	5 (11.11)	1 (6.25)	2 (14.3)
	More than 15 years	8 (17.78)	2 (12.50)	–

Survey 1: other n=3; physician associate, advanced nurse practitioner.

Survey 2: other n=1; advanced clinical practitioner.

### NoMAD surveys

Table 3 presents responses from surveys 1 and 2 and the paired surveys and is narratively synthesised below.

**Table 3:** NoMAD surveys 1 and 2 and paired response

NPT constructs	NoMAD survey questions	Percentage (%) strongly agree/agree			
		Survey 1 (n=45)	Survey 2 (n=16)	Survey 1 paired (n=14)	Survey 2 paired (n=14)
Coherence	I can see how the NAT-C differs from usual ways of working	87	81	86	79
	I understand how the NAT-C affects the nature of my own work	93	94	100	71
	I can see the potential value of the NAT-C for my work	98	81	100	79
Cognitive participation	Key people may be needed to drive the NAT-C forward and get others involved	95	94	86	93
	I believe that using the NAT-C would be a legitimate part of my role	95	69	100	71
	I’m open to working with colleagues in new ways to use the NAT-C	100	81	100	86
	I am likely to support the use of the NAT-C	95	81	93	79
Collective action	I can easily integrate the NAT-C into my existing work	67	37	79	36
	The NAT-C may disrupt working relationships	7	6	7	7
	The training provided is sufficient to enable staff to implement the NAT-C	86	94	85	85
	Sufficient resources are available to support the use of the NAT-C	64	56	71	50
	Management will adequately support the use of the NAT-C	75	37	93	43
Reflexive monitoring	Practice staff will agree that the use of the NAT-C is worthwhile	67	62	79	64
	Feedback about the NAT-C can be used to improve it in the future	98	100	93	21
	I can modify how I work with the NAT-C	82	81	71	86

NAT-C, Needs Assessment Tool-Cancer; NoMAD, Normalisation MeAsure Development Questionnaire; NPT, normalisation process theory.

#### Coherence

Most clinicians understood how the NAT-C could influence their work and strongly agreed/agreed on the potential positive value of the NAT-C.

#### Cognitive participation

Nearly all clinicians strongly agreed/agreed that key people would be needed to drive the NAT-C forward. Similarly, nearly all believed that the NAT-C was a legitimate part of their role, and they would support its use, although the proportion agreeing was slightly less following use of the NAT-C.

#### Collective action

Most clinicians strongly agreed/agreed that the training provided was sufficient. In Survey 2, they felt less sure that the necessary resources and management support would be available.

#### Reflexive monitoring

In Survey 1, most strongly agreed/agreed that feedback about the NAT-C could be used to improve the tool; however, following the experience of using the tool, few maintained this view. Clinicians felt that they could modify how they worked with the NAT-C and largely remained positive that practice staff would agree that use of the NAT-C is worthwhile.

Overall, the results for the paired responses demonstrated that while participants’ views remained mostly positive, there were more concerns relating to resources and management support in Survey 2. Interestingly, once clinicians had experience of using the NAT-C, they did not feel it needed significant alteration and wouldn’t benefit from further feedback.

### Clinician and key stakeholder interviews

#### Interviews

Interviews were conducted from August 2021 to December 2021 online. We paused during a national healthcare system delivery change, with further interviews conducted from December 2022 to October 2023. Interviews lasted on average 41 min (range 30–52 min).

#### Interview participant characteristics

The characteristics of the 16 participants (eight study site clinicians, eight key stakeholders) are summarised in table 4.

**Table 4:** Clinician and key stakeholder interview participant characteristics

Clinician interviews (n=8)	
Profession	Salaried GP=3 GP partner=5
Gender	Male=5 Female=3
Years of experience	<5 years=1 5–10 years=3 10–15 years=2 15–20 years=2
Specialty role: yes/no	Cancer lead: yes=0; no=8 Palliative care lead: yes=2; no=6
Key stakeholder interviews (n=8)	
Role description	Various roles including Macmillan Lead, clinical advisors and regional roles; living with and beyond cancer, Cancer Alliance*
Gender	Male=3 Female=5
Location	UK

* Summarised only to preserve anonymity.

GP, general practitioner.

Five themes were developed from the clinician and key stakeholder interviews (see table 5 and online supplemental file 5 participant quotes).

**Table 5:** Clinician and key stakeholder interviews key themes, subthemes and NPT constructs

Theme	Subthemes	NPT constructs
1. The perceived value of NAT-C The NAT-C was seen as valuable for patient care and aligned with good cancer care; this is a major driver for implementation	Sensitive, awkward questions/permission to ask and probe Beyond unmet need; patients feel ‘seen’ as a person: person-centred For other diagnoses and long-term conditions Over other tools, fits with consultation style skills what GPs want to do Across patient stages of cancer journey, one tool applicable from diagnosis for cancer care reviews, after secondary care cancer treatment and end of life Information sharing with the team and fostering teamwork Training framework for cancer care Patient benefit through supporting self-management and more appropriate help-seeking behaviour The systematic approach as a drive to reduce inequalities; all patients are invited	Coherence *Sense-making work*
2. Champions are key Enthusiasts are important for implementation in practice, regional and national levels	Enthusiasts, interest or GP role at practice in cancer or palliative care, networks—PCN to drive implementation, leadership role	Cognitive participation *Relational work*
3. Effectiveness evidence, while important, has a limited impact on implementation Research evidence was seen as important, but influences implementation indirectly through policy and resource initiatives	Evidence of clinical benefit is hard to demonstrate, current tools evaluated in quality improvement projects, or trials reporting process outcomes only Evidence influences NICE guidelines, which influence clinical practice, but mainly through policy drivers with financial incentives such as QOF Value (personal experience of patient benefit) trumps effectiveness evidence in terms of individual clinician implementation	
4. Adequate resources are fundamental for implementation beyond the practice level Policy and evidence-based clinical guidelines are needed to drive regional and national implementation, but cannot be done without sufficient resources	Capacity of the primary care workforce in the current context: GP staffing pressures individual capacity with nihilism—don’t want to find things, can’t fix and don’t go looking; lack of time to deliver and train Financial resources (eg, QOF), but danger of tick box exercise and poor-quality NAT-C completion, directory of services to refer to (eg, community, charity links) Potential use of existing resources: role of PCNs in the use of current systems infrastructure already existing in community setting, charities; triage and use of skill mix, for example, nurses, advanced clinical practitioners, non-medical cancer care coordinator, link worker; use of PCN could reduce workload on individual practices—network level, national level	Collective action *Operational work*
5. NAT-C practicalities The training was adequate and informative, but it would need robust functional IT systems to support implementation. The tool could be simplified	Training was good and adequate, video consultation appreciated and remembered Learning curve is usual when using a new tool, irrespective of the training received, use of an introductory spiel, clunky, understanding a tool to support discussion, initially asking all domains and refining delivery with experience IT systems integration; accessibility, visibility, easy to find and use, prompts and reminders for information sharing NAT-C tool template modifications; simplification, embedding current systems such as Ardens, context of delivery; adaptation for telephone to address carers	Reflexive monitoring *Appraisal work*

PCN: groups of general practices working together with other community health services.

QOF: a central incentive initiative in UK primary care.

GP, general practitioner; IT, information technology; NAT-C, Needs Assessment Tool-Cancer; NICE, National Institute for Clinical Effectiveness; PCN, primary care network; QOF, quality outcomes framework.

##### Theme 1: the perceived value of NAT-C

The NAT-C was seen as valuable in patient care and aligned with good cancer care; this is a major driver for implementation. Both clinicians and key stakeholders thought the tool would be of value at different stages of the cancer journey. Views varied as to when it would be most applicable: at diagnosis, after completing cancer treatments or for palliative care management.

C1 … it is a really helpful tool to have, …. at any stage of cancer, but I think particularly more towards the end palliative care stages

Proactive assessment of concerns was viewed as valuable to support people with cancer, and especially people with multimorbidity. The possibility that proactive assessment of concerns may reduce health service costs was raised as a health economic rationale for implementation.

K6 Well, I think a lot of people probably are going to have more comorbidity, aren't they, certainly in our population? Erm, and so it’s maybe addressing their cancer needs alongside their other chronic disease needs

K5 … but also maybe they've (the patient) got an increased ability to self-manage …, so that all speaks very loudly to, erm, the people you'd be trying to convince in how to—how to put this in as business as usual

Clinicians emphasised the value of the NAT-C over similar tools in fostering a person-centred relationship. They felt it went beyond identifying unmet needs and encouraged probing with sensitive questions using a consultation style consistent with their core values and skills, supporting their personal development. There was recognition that every identified unmet need might not be ‘fixable’, but that the assessment was itself therapeutic.

C3 It was nice for them (patient trial participant) to have the issues that they raised, erm, acknowledged, ‘cos, like you say, part of, I think, good medical care is … empathy, which is, you know, the basis of the placebo effect, which is extremely powerful, and so, you know, the fact that the message is received, that your GP cares about this, erm, I got the sense that that was, erm, valued

Stakeholders identified the potential use of the NAT-C as a training framework and an important route to share information with the wider team.

K8 So—so this tool—almost all this, you know, the tool almost becomes the framework to have a—a training session that’s about the—why—why tailored care, whole person care, that—that recognises these multiple elements is—is—is—is appropriate

K8 when I was a palliative care lead, trying to design a tool that was probably a bit like this, …. That was, ‘how do we collect in one place the information that helps us to understand from a whole person perspective what’s going on here, and—and share that information with the team, so everybody can come in and quickly see it and then know what their action point is’. So there’s something about a tool like this that’s really valuable for team support

##### Theme 2: champions are key

Enthusiasts, such as clinicians with a special interest in cancer or palliative care, were considered essential for the implementation of the NAT-C in practice, regional and national levels. Also, administrative support was needed to identify, invite and triage patients, especially if implementation was to take place beyond the practice level. Scaling up would raise awareness and increase impact beyond that of single practice implementation.

K3 So it’s a combination of someone championing it clinically, I would say; some admin support; and then maybe (name) or somebody like that that brings them into those appointments

C8 … if it’s a national thing, more people are doing it. I think the impact will be more significant, and then the discussions there, people are talking about it. If it’s only one practice, there’s less discussion, less awareness, less engagement…

##### Theme 3: effectiveness evidence, while important, has a limited impact on implementation

Effectiveness and cost-effectiveness evidence were seen as important, but while some individual clinicians were evidence-based practitioners, more generally, evidence appears to influence implementation indirectly through policy and resource initiatives. Inclusion in guidelines such as NICE would raise awareness and publicise the NAT-C, but implementation wouldn’t necessarily follow unless clinicians see benefit in their own practice. While cost-effectiveness and clinical guidelines were important for commissioners, some clinicians felt overwhelmed by the volume of NICE recommendations and deemed observing patient-related impact as the motivation for implementation. The complex budgetary system of healthcare led to some scepticism regarding a cost-effectiveness argument.

C5 I think, there has to be like a demonstrable benefit to the GP. I don't believe that needs to be financial, but … a definite benefit to ‘why am I going to sit down and learn about this new form and start to use it?

K1 There’s so much of it (NICE guidance), you can't keep up with it and ….So NICE saying something doesn't necessarily influence us in primary care

##### Theme 4: adequate resources are fundamental for implementation beyond the practice level

Policy and evidence-based clinical guidelines are needed to drive regional and national implementation, but they need sufficient resources. Clinicians and stakeholders perceived that the clearest route to implementation was monetary incentives. However, many were concerned that this would risk degrading the quality of the NAT-C to a ‘tick box’ exercise, thereby making patient benefit unlikely.

C4 if it’s on the QOF, the GP practices will do it, erm, 100%, and GPs …have systems set up in their practice to – to do that, so if you're going to try and get it – get GPs to do it, you have to get through the QOF…

K1 …you can literally just tick the box that says you've done it and get the payment. There really isn't an incentive to try and do it well

The lack of capacity of primary care was a key context that would need to be overcome for NAT-C implementation, as current services were overstretched with extreme pressure on GPs.

C3 … the elephant in the room here is lack of resources, you know? … the NAT-C is identification of unmet need, and in primary care … we can't meet identified need

The current lack of capacity to engage with new interventions systematically was echoed by stakeholders. Primary care was seen as the ideal setting to address the holistic needs of people with cancer. However, being overwhelmed with the current pressures was seen as a major barrier, with additional staffing and time being fundamental to any implementation strategy.

K1 … We know it’s great for patients. But we still have very limited capacity. Something’s got to give. So I think yes, if you can play the card that, actually, if you do this well, you'll get a lot of more satisfaction for your job; the patients will be more satisfied; it will reduce complaints, which would then reduce workload for some teams who have to deal with complaints; it will reduce, you know, attendances, moving forwards, and it reduces their chances of hospital admission, and it, you know, prolongs their life or whatever evidence it is

Pressures on primary care practice meant that GPs and key stakeholders alike felt NAT-C delivery could be delegated within the clinical team, for example, nurses, or non-clinical staff—at least for initial triage. However, some GPs were clearly torn between the need to delegate work because of time pressures and wanting to incorporate it into *their* role.

C2 …we’re the holistic, whatever, you know, and often we do know a lot more about their background and their family situation if they've been at the practice a long time. So, I think it is good for us to be involved. … Erm, I don't like sound like it’s not my responsibility, but equally, as I said, it’s pressures and time resources, isn't it?

Finding the time to conduct a NAT-C consultation (even 15 min more than the standard 10-minute appointment), or complete training (even an hour, or less) was a concern. Triage was identified as a way of targeting the NAT-C to those with the most unmet need.

C4 …most of my assessments were between 20 and 25 minutes long, so it’s quite a big piece of work…. Erm, so I think there needs to be a way of selecting those that that need it most and then identifying those

Similarly, key stakeholders talked about restricted time in the context of exhausted clinicians. They suggested that primary care networks (local groups of GP practices) and integrated care systems (regional commissioning bodies) could foster capacity to innovate, along with the existing infrastructure—including linking with voluntary and third sector organisations. A systems approach is needed with regional leadership from service commissioners and efficiencies from GP practices working together, for example, a primary care network-based cancer coordinator working across several GP practices for the identification of patients and initial triage.

K8 They're—they're so busy running around the (hamster) wheel that—… they haven't got time for—headspace for anything else. So if we're going to introduce a new tool—yet another new tool—into that context, it’s going to have to be done in a - in a setting that’s big enough to have the potential capacity to do it, and I would suggest that that’s going to be probably PCN (primary care network) level as a minimum, … groups of practices who - who are working together and potentially therefore able to—to—to find the additional capacity they need to innovate around this

K4 I think you do need that leadership at the ICS (Integrated Care System) and commitment to a whole system approach that’s going to respond to need ….

Workforce pressures led some GPs to voice a sense of nihilism that “you don’t go looking for things for fear you might find something that you can’t treat”, despite most recognising the therapeutic benefit of acknowledgement, even if the issue cannot be resolved.

C3 … to be honest, I wouldn't want that training, because what it would identify is a load of need that I wouldn't feel that I could meet within the constraints of the current system

##### Theme 5: NAT-C practicalities

Clinicians deemed the NAT-C training as good, informative and adequate. Memory of the training session was variable, due to the length of time between training and delivery of the NAT-C, but clinicians mostly recalled and appreciated the video consultation.

C5 I don't have anything negative to—to say about the—about the training…., because when you look at the NAT-C tool, it can look a bit tick-boxy, which is obviously something that we don't like and, erm, generally, erm, is a barrier to—to a good consultation, rather than a kind of facilitator. But so it was useful to see the video of the way it was envisaged it would be used, in a way that was less structured…

Some needed to use the NAT-C after training before they felt comfortable, but this was viewed as an expected learning curve. Some clinicians expressed that it felt ‘clunky’, although they understood that the tool aimed to support and facilitate conversation rather than to be used as a checklist.

C4 …—you get better the more you do, the better you get at it, and you can have all the training you like, actually, until you start delivering the intervention

IT systems and the NAT-C electronic template configuration were highlighted by clinicians and key stakeholders as important. Most felt that the template was straightforward to use but would need more integration, making sure that the tool was easy to find, use and included prompts and reminders for information sharing. Suggestions for improvement included streamlining some questions, particularly around the family carers, and allowing free-text boxes for each domain. Clinicians suggested embedding in systems already in use to address clinicians’ negative attitudes towards templates and encourage NAT-C use over others available.

K7 I would say it needs to be, erm, template—driven, so you use to be a good IT—IT, erm, and the template needs to be easy to—easy to navigate and not overly busy, erm, because that itself will—will push people away and it needs to—… be relatively time—efficient to be able to utilise it

S2 R10 Easy to use, good prompts. I did modify how I used it. … The carer bit never quite worked—mainly as the carer was often not present during the interview, so most information was second hand

### Synthesis of all findings

Synthesised findings are presented in tables6  7.

**Table 6:** Synthesised main findings of NAT-C training and intervention fidelity

Aims	NAT-C intervention training and fidelity	NoMAD surveys 1 and 2 (percentage (%) agree)	Interviews: clinicians and stakeholders	Synthesised findings
Adequacy of NAT-C training	21 GP practices, n=53 clinicians **Clinicians trained per practice** Mean (SD) 2.5 (1.36). GPs; n=35 (66%) Practice nurses; n=13 (24.5%) **Days between training and first NAT-C appointment** Mean (SD) 105.68 (47.93) Median 97.00 (range 46.00–230.00)	The training provided is sufficient to enable staff to implement the NAT-C Survey 1 (n=45) 86% Survey 2 (n=16) 94% Paired surveys 1 and 2 (n=14) 85–85% unchanged **Open text responses** Training informative and helpful	**Informative adequate training** Good, adequate, video consultation appreciated and remembered However, the memory of training was poor except for the training video	Clinicians deemed the training adequate and informative. There were no negative impacts from having the training online The time from training to delivery of the first NAT-C appointment was over 3 months, and some clinicians only recalled the video consultation in any detail. They deemed the video to be an effective training tool
NAT-C intervention fidelity*	Total NAT-Cs delivered n=360/376 (96%) **Length of consultation** (n=360) Minutes: mean (SD) 24.4 (9.18) Median 24.0 (range 8.0–60.0) IQR (20.0–30.0)	I can see how the NAT-C differs from usual ways of working Survey 1 (n=45) 87% Survey 2 (n=16) 81% Paired surveys 1 and 2 (n=14) 86–79%	Risk of tick box A bit clunky Aligns with consultation style Challenge to get everything done in one consultation Learning curve when using a new tool, irrespective of the training received	The tool had high fidelity, with nearly all participants receiving a NAT-C consultation. This was consistent with survey and interview data confirming that clinicians felt the tool was relevant and was a prompt that aligns with usual consultation style, and is not a tick box Use in practice—learning curve And concerns that it *could* be degraded in practice to a tick box, especially given time pressures

* NAT-C intervention fidelity item completion not recorded.

GP, general practitioner; NAT-C, Needs Assessment Tool-Cancer; NoMAD, Normalisation MeAsure Development Questionnaire.

**Table 7:** Synthesised main findings, possible mechanisms of action and issues with implementation

Aims	NAT-C intervention training and fidelity	NoMAD surveys 1 and 2 (percentage (%) agree)	Interviews: clinicians and stakeholders	Synthesised findings
Possible mechanisms of action for implementation in practice	Apparent unmet need: Baseline trial questionnaire: % participants with at least one moderate-severe unmet need at baseline=52%; expressed need On NAT-C**—**at least one need (n=232 (64.4%)) Referrals NAT-C (n=360). No: 310 (86.1%) Yes: 50 (13.9%) Highest action taken per NAT-C section Direct management n=258 (71.7%) Managed by another team member (n=61 (16.9%))	I can see the potential value of the NAT-C for my work Survey 1 (n=45) 98% Survey 2 (n=16) 81% Paired surveys 1 and 2 (n=14) 100–79% I believe that using the NAT-C would be a legitimate part of my role Survey 1 (n=45) 95% Survey 2 (n=16) 69% Paired surveys (n=14) 100–71% I can easily integrate the NAT-C into my existing work Survey 1 (n=45) 67% Survey 2 (n=16) 37% Paired surveys 1 and 2 (n=14) 79–36% Feedback about the NAT-C can be used to improve it in the future Survey 1 (n=45), 98% Survey 2 (n=16) 100% Paired surveys 1 and 2 (n=14) 93–21% Practice staff will agree that the use of the NAT-C is worthwhile Survey 1 (n=45) 67% Survey 2 (n=16) 62% Paired surveys 1 and 2 (n=14) 79–64% I can modify how I work with the NAT-C Survey 1 (n=45) 82% Survey 2 (n=16) 81% Paired surveys 1 and 2 (n=14) 71–86% I’m open to working with colleagues in new ways to use the NAT-C Survey 1 (n=45) 100% Survey 2 (n=16) 81% Paired surveys 1 and 2 (n=14) 100–86%	**Enthusiasts/champions** at practice, regional and national levels; at practice in cancer or palliative care, networks—PCN to drive implementation, leadership role **Potential value of NAT-C** Patient value acknowledged and seen as person centred Fits consultation style and skills Sensitive awkward questions/permission to ask Potential value for other diagnoses and long-term conditions Value over other tools Patient stage of cancer journey Information sharing with the team Training framework Patients benefit from self-managing Applicable to other chronic conditions Could be conducted by other primary care team members and use the skill mix already in the team **Policy and resourced initiatives implementation** Usual route Financial incentives Risk of tick box exercise Requires champions, value and resources **IT and NAT-C template** IT systems integration; accessibility, visibility, easy to find and use, prompts and reminders for information sharing **Evidence influences implementation indirectly through policy and resourced initiatives (QOF**) Evidence of effectiveness NICE guidelines Clinicians' value trumps effectiveness	Surveys and interviews strongly endorse the benefit of champions at local, regional and national level to drive the NAT-C forward and get others involved The unmet need was clear from the quantitative data. The tool was seen as valuable, beneficial to patients and aligned with the clinician’s perceived role in primary care. Most could see how the NAT-C differed from usual ways of working, but it was seen as beneficial and aligned with their consultation style and could be adapted for other chronic disease management Some clinicians recognised the benefit of acknowledgement of issues that could not be ‘fixed’ Clinicians and key stakeholders considered that NAT-C delivery was flexible and could be conducted by other team members (clinical or administrative) Research evidence was important, but apart from some very evidence-based clinicians, personal experience of benefit was most important for individuals, and the evidence base drove implementation indirectly through policy/finance incentives and clinical guidelines
Possible issues with implementation in practice	See above are level of unmet need	Key people may be needed to drive the NAT-C forward and get others involved Survey 1 (n=45) 95% Survey 2 (n=16) 94% Paired surveys 1 and 2 (n=14) 86–93% Sufficient resources are available to support the use of the NAT-C Survey 1 (n=45) 64% Survey 2 (n=16) 56% Paired surveys 1 and 2 (n=14) 71–50% Management will adequately support the use of the NAT-C Survey 1 (n=45) 75% Survey 2 (n=16) 37% Paired surveys 1 and 2 (n=14) 93–43%	Clinicians perceived that study participants had few unmet needs, questioning the need for routine application **Resources and network approach** to extend beyond practice level implementation **Risks and challenges** Capacity of primary care Context pressure and loss of continuity GP staffing pressures Individual capacity Time to deliver and train Financial resources Nihilism conflict with time/capacity to complete adequately/quality **IT and NAT-C template** NAT-C tool template simplification and modifications to address carers (context of delivery) Research evidence of clinical benefit is hard to demonstrate. Current tools evaluated in quality improvement projects, or trials using process outcomes only. Resources and funding are crucial	Clinicians perceived that patients had few unmet needs, yet trial data show this perception to be unfounded (see facilitators) Some clinicians were worried about raising hope regarding issues they felt they could not help (eg, fatigue). However, the trial findings showed that fatigue was improved in the NAT-C arm compared with the usual care arm, and interview data confirms many could see the benefit of acknowledging issues even if there wasn’t a ‘fix’ There was a strong emphasis on the need for resources, support from management and the use of established networks—without which even an evidence-based intervention, recommended in guidance, is unlikely to be implemented beyond local practices Interviews identify current limited capacity in primary care, with staffing, finances, loss of continuity, exhausted clinicians and time constraints as key challenges to effective implementation

GP, general practitioner; IT, information technology; NAT-C, Needs Assessment Tool-Cancer; NICE, National Institute for Clinical Effectiveness; NoMAD, Normalisation MeAsure Development Questionnaire; PCN, primary care network; QOF, quality outcomes framework.

The NAT-C consultation occurred in nearly all patient-participants registered with intervention practices. NAT-C assessments were delivered as a consultation, and not ‘ticking boxes’– although the risk of such an approach was recognised.

Clinicians found training useful and informative, and online delivery was acceptable. Some clinicians recalled the training poorly, apart from the video consultation, which was deemed particularly helpful. Despite some clinicians having considerable time between training and delivery of the first NATC, this did not appear to adversely impact their ability to do so. A learning curve was evident from the interview findings, regardless of the training.

Clinicians felt the NAT-C was valuable and aligned well with their role, a major driver to implementation. They perceived that most patients had few unmet needs, questioning the need for routine implementation, but this perception was not consistent with trial data, where nearly two-thirds of NAT consultations identified unmet needs, of which over three-quarters required action.

Enthusiasts or champions at the local, regional and national levels are necessary to drive NAT-C use, but insufficient resources would block implementation of a valuable, evidence-based intervention.

Management support was important. Limited capacity in the current NHS primary care workforce crisis, with low levels of staffing, limited finances, loss of continuity and time constraints, were presented as key barriers, with innovations afforded low priority by management. Use of established infrastructure with shared staff, for example, primary care networks, was a potential amelioration within current resources. Current wider clinical and administrative teams could be used rather than expecting GPs to deliver, although GPs felt torn at being unable to conduct a consultation that aligned so well with their values. The escalating primary care workforce crisis over the course of the trial could be seen in the paired survey data, with far fewer clinicians agreeing that the NAT-C could easily be integrated into their existing work.

Although interviewees acknowledged the importance of effectiveness and cost-effectiveness data, this was viewed by most as driving change through clinical guidelines and other incentives, rather than convincing individual GPs directly, where personal experience of patient benefit, resources and champions were stronger motivations. However, they acknowledged that the evidence base contributed to a risk of public censure (or recommendation for good quality care) through the Care Quality Commission (national regulator of health and social care), and an increase in financial resources through the QOF. This latter aspect illustrated tensions between the need for financial incentives and the risk of a ‘tick box’ culture and poor-quality assessments in a context where insufficient resources limited the ability to provide proactive care.

## Discussion

In this process evaluation of the intervention arm of a cRCT, NAT-C delivery fidelity was high, with nearly all intervention-practice participants receiving an NAT-C-guided consultation. The NAT-C assessment helped identify and plan action for unmet needs in nearly two-thirds of intervention practice participants, despite a perception by clinicians that most had few needs. This supports the use of a systematic approach to ‘uncover’ otherwise hidden issues; clinicians may not ‘see’ significant problems, and the patient may not volunteer them without prompts.^18 19^ Clinicians found the training video particularly useful and viewed the NAT-C as beneficial for patients and aligned well with their own values. This alignment and perceived benefit were seen as a major driver of implementation, alongside champions. However, insufficient resources and lack of management support (in the context of an unfolding major NHS primary care workforce crisis over the course of the trial) were seen as serious issues that would block implementation. Use of established infrastructure with shared staff, using wider clinical and administrative teams, could ameliorate this challenge to a certain extent. Effectiveness and cost-effectiveness data were viewed by most as helpful in driving change, but indirectly through clinical guidelines, financial incentives and national regulatory bodies, rather than convincing individual GPs directly. Tensions between the need for financial incentives and the consequential risk of a ‘tick box’ culture with poor-quality assessments were apparent.

### Comparison with the literature

The barriers and facilitators of implementing change involving complex interventions are well documented in primary care.^5^ Consistent with our findings, the biggest barrier to implementation is a lack of resources. A systematic review of reviews of strategies designed to implement change in primary care settings^20^ found strategies mainly directed at professionals, with few targeting organisational or wider context change. Those targeting a single category (eg, professional education) improved practice by a small amount only (2–9%). Multifaceted strategies did not deliver better results; a finding confirmed by another systematic review of reviews comparing single versus multiple interventions,^21^ although the distinction between single and multiple strategies is questionable.^22^ Despite strong views among our study participants that local champions were key to implementation, Lau and colleagues found that printed educational materials and local opinion leaders were the least effective strategies.^20^

Harvey and Kitson^22^ argue that an approach to implementation using simple categories (professional, organisational, financial, etc) that does not involve a detailed understanding of context will fail. Our findings support this observation. Clinicians and stakeholders viewed the NAT-C as strongly aligning with primary care clinicians’ values and a legitimate part of their role that would benefit patients, helping them deliver the level of care to which they aspire (*coherence, cognitive participation*). The training was viewed as helpful and short (only one <1 hour session), the NAT-C assessment took on average only 10–15 min longer than a standard consultation, and one cancer review was already included in the QoF financial incentive scheme. This positive attitude was reflected in the high adherence and fidelity in NAT-C delivery. However, during the study, the crisis in NHS primary care service delivery deepened; disillusionment in primary care practice,^23^ COVID-19 pandemic-related challenges^24 25^, medical staff mix changing (GP partner decreases: 70.1% in 2017 to 61.1% in 2022; salaried posts increases: 25.5% in 2017 to 35.5% in 2022),^26^ alongside increases in video- or phone consultations, Brexit-related staff recruitment issues, NHS regional reorganisation, increasing clinical pressures on primary care and the NHS in general post-pandemic). Burnout is one of the most cited reasons for UK GPs leaving the profession; a higher proportion than in other specialties (43% vs 22%).^27^

This context was reflected in a stark concern (Survey 2 and the interviews) that a lack of resources and a lack of confidence in organisational support would block innovation despite recognising the NAT-C’s positive features. Although most clinicians could see how the NAT-C could be incorporated into their workday (*reflexive action*), the predominant voice was that the *collective action*, including at the level of other practice staff, required to embed the NAT-C into routine care, was inadequate. It is remarkable, therefore, that despite this context of a tired and perhaps burnt-out workforce, there were still those able to suggest ways that the collective *could* implement the NAT-C within existing or possible infrastructures, at least locally. However, the message is clear; any implementation strategy must address the broader context of workforce pressures and NHS primary care service delivery models and embrace the complexity to scale up to regional or national implementation.

There was ambivalence about the role of financial incentives, clinical practice guidelines and regulation. This relates to worries that effective interventions would be poorly implemented (ticking the boxes), reducing their effectiveness, but still increasing staff time commitment. The impact of financial incentive schemes in UK primary care has been questioned; Allen *et al* found that while the quality of care was increased at 1 year, by 3 years the impact on clinical change was similar to the underlying trend prior to implementation.^28^

Evidence of effectiveness, and particularly cost-effectiveness, was important for implementation, but our participants had little confidence that evidence alone would drive widespread uptake of the NAT-C. In the context of a complex funding model of secondary and primary care, there was scepticism that even a cost-effective intervention would be implemented. Therefore, although our main study indicated a high probability of cost-effectiveness,^8^ consistent with the published literature,^5^ even if the NAT-C was included in national guidelines, for example, NICE, without accompanying sustainable and sufficient funding to resource training and staff, then national implementation would struggle. Philanthropic, or other short-term NHS service development funding, was seen as a good way to develop improvement initiatives and resources, with education and training, network support staff and clinical leaders. However, sustaining such services if funding was withdrawn was difficult, even if NHS cost-savings could be demonstrated; long-term financial support and reconfiguration are needed.

### Implications for practice, research and policy

Clinicians could see a place for NAT-C assessment soon after diagnosis, at the end of hospital treatment, in cancer recurrence or deterioration of health status, and as part of end-of-life care. The NAT-C is designed so that any member of the primary care team, including administrative triage workers, could conduct the initial screening with the NAT-C, directing as needed to other members of the team. Network-wide systems could be put in place (eg, using network cancer coordinators) could be used for this purpose.

Future research is needed to confirm or refute the hypothesis-generating findings from the main trial, using a primary endpoint of 6 months. Studies should be hybrid effectiveness-implementation studies. Further refinement and evaluation of the tool for long-term conditions (generic) could be useful in chronic care.

Policymakers and clinical guideline developers should be aware of the NAT-C and its developing evidence base. Service commissioners should look to the redistribution of NHS funds for interventions shown to be cost-effective; recent rhetoric is promising, but yet to be seen in practice.^29^

### Strengths and limitations

This process evaluation used a mixed-methods synthesis underpinned by NPT, which added value to the understanding of potential implementation issues. Paired surveys and interviews stretching over the course of the study enabled the impact of changing context to be seen.

Due to recruitment difficulties and mounting work pressures, we could not recruit to our target interview sample or purposively sample as intended.^9^ The viewpoint of other clinicians, such as practice nurses, who do not have the same consultation skills training as GPs in the UK, is unknown, as we were unable to recruit them. They may have found it more difficult to incorporate the NAT-C as part of a consultation rather than completing it as a questionnaire. However, we generated rich data, enhanced by our wider stakeholder interviews, which gave us depth of insight into the national and regional contexts.

## Conclusions

This NPT-informed process evaluation of the intervention arm of a clinical trial showed that the NAT-C was seen as a relevant, valuable tool for patient benefit in primary care cancer care that could be used as a legitimate part of the GP’s role. However, an emerging wider context of serious workforce pressures across the NHS, and particularly in primary care, meant that unless adequate and sustained resources (funding, training and staff) were provided, implementation in anything other than a loco-regional context would be unlikely.

## Supplementary material

10.1136/bmjopen-2025-113686 online supplemental file 1

10.1136/bmjopen-2025-113686 online supplemental file 2

10.1136/bmjopen-2025-113686 online supplemental file 3

10.1136/bmjopen-2025-113686 online supplemental file 4

10.1136/bmjopen-2025-113686 online supplemental file 5

## Data Availability

Data are available upon reasonable request.
