# Supplementary material for: Mixed methods process theory evaluation to explore the implementation issues of the Needs Assessment Tool-Cancer (NAT-C) in primary care for people with cancer
Source: BMJ Open. 2026 Apr 8;16(4):e113686. doi: 10.1136/bmjopen-2025-113686 (PMC13064150; doi:10.1136/bmjopen-2025-113686)
Supplement: online supplemental file 1 [file bmjopen-16-4-s001.pdf]

**Supplementary file 1**

**NAT-C Training resources**

*Essential resources for NAT-C training*

- The Needs Assessment Tool-Cancer for upload to computer systems as a Document(Microsoft Word Version)
- Templates for SystmOne or EMIS
- Instructions on how to upload the Templates (SystmOne/EMIS)
- Screenshots of how the NAT-C will look on SystmOne/Emis

*CANAssess2 training slides*

- Welcome to the Needs Assessment Tool - Cancer (NAT-C) training
- What is the CANAssess2 study?
- Rationale for the study
- Evidence supporting use of a systematic approach to symptom assessment (as opposed to open questioning)
- What is the NAT-C?
- How to use the NAT-C
- Video of a real-life patient consultation
- Frequently Asked Questions
    - Can I complete a NAT-C consultation in 20 minutes?
    - What if I can't complete the whole form during a consultation?
    - I've identified a problem, but there is no service to refer to?

      Why is the NAT-C only for cancer patients?
