# Supplementary material for: Mixed methods process theory evaluation to explore the implementation issues of the Needs Assessment Tool-Cancer (NAT-C) in primary care for people with cancer
Source: BMJ Open. 2026 Apr 8;16(4):e113686. doi: 10.1136/bmjopen-2025-113686 (PMC13064150; doi:10.1136/bmjopen-2025-113686)
Supplement: online supplemental file 2 [file bmjopen-16-4-s002.pdf]

Supplementary file 2 Topic guides for clinician and key stakeholder interviews

Interview Schedule: GP practice staff

At the start, the researcher will introduce self, summarise what will happen and ensure participants have provided consent to interview/group and being recorded.

Interview and focus group discussions will be semi-structured, guided by the following domains, starter questions and prompts.

Study training

Question: Do you feel that study training sessions (face to face / online) enabled you to effectively use the NAT-C?

- What did you find useful?
- What was less useful?
- What other information would you have liked?

The Needs Assessment Tool: Progressive Disease Cancer (NAT-C)

Question: How did you find using the NAT-C to guide a patient consultation?

Question: How does the NAT-C differ from your usual practice?

Question: What should initiate a NAT-C review?

Potential implementation of the NAT-C in to general practice

Question: Tell me about your experience of implementing the NAT-C within your practice?

- What has worked well?
- What are the challenges with regard to implementing this in routine practice?
- How can these be overcome?
- How could the NAT-C be incorporated into everyday practice?

Interview Schedule: key stakeholders

At the start, the researcher will introduce self, summarise what will happen and ensure participants have provided consent to interview/group and being recorded. Prior to commencing focus groups, participants will be introduced to each other and each participant will be asked to confirm that they are happy to proceed with the focus group. Participants unwilling to proceed will be free to leave the focus group and offered the opportunity to take part in a separate interview if they wish. Participants will be reminded that they have provided informed consent for interviews/focus groups to be audio-recorded and that any information they provide will be handled confidentially, in accordance with the Data Protection Act 2018.

Interview and focus group discussions will be semi-structured, guided by the following domains, starter questions and prompts. The interviewer will explain the purpose of the NAT-C ahead of the following questions.

The Needs Assessment Tool: Cancer (NAT-C)

Question: If effective, do you think that the NAT-C could be implemented in to general practice?

Question: What place, if any, can you see for the NAT-C in routine primary cancer care?

- How might this fit with other initiatives?

Question: How could the information about patterns of unmet need and referrals resulting from a NAT-C consultation be used in service commission, planning and delivery?

Potential implementation of the NAT-C in to general practice

Question: What do you see as the challenges of implementing the NAT-C in to general practice?

- How can these be overcome?
