# Supplementary material for: Mixed methods process theory evaluation to explore the implementation issues of the Needs Assessment Tool-Cancer (NAT-C) in primary care for people with cancer
Source: BMJ Open. 2026 Apr 8;16(4):e113686. doi: 10.1136/bmjopen-2025-113686 (PMC13064150; doi:10.1136/bmjopen-2025-113686)
Supplement: online supplemental file 3 [file bmjopen-16-4-s003.pdf]

Supplementary File 3

Box 1 Description of Normalisation Process Theory constructs in relation to potential implementation of the NAT-C

**NPT constructs**	**NPT construct description**
Coherence	***Sense making work:*** How do clinicians understand and plan the activities that need to be accomplished to put the NAT-C into practice?
Cognitive participation	***Relational work:*** How do clinicians engage and work together to create networks of participation and communities of practice around the NAT-C?
Collective action	***Operational work:*** How do clinicians work individually and together to enact the NAT-C?
Reflexive monitoring	***Appraisal work:*** How do clinicians work individually and together to appraise the NAT-C?

Derived from May (2013) (16)
