# Supplementary material for: Mixed methods process theory evaluation to explore the implementation issues of the Needs Assessment Tool-Cancer (NAT-C) in primary care for people with cancer
Source: BMJ Open. 2026 Apr 8;16(4):e113686. doi: 10.1136/bmjopen-2025-113686 (PMC13064150; doi:10.1136/bmjopen-2025-113686)
Supplement: online supplemental file 4 [file bmjopen-16-4-s004.pdf]

Supplementary file 4 Intervention delivery, uptake and fidelity

Table 1 NAT-C intervention fidelity

	**Hull (n=99)**	**Leeds (n=91)**	**Sheffield (n=133)**	**Sunderland (n=53)**	**Total NAT-C (n=376)[1]**
**NAT-C Intervention delivered**					
Yes	96 (97.0%)	85 (93.4%)	126 (94.7%)	53 (100.0%)	360 (95.7%)
No	3 (3.0%)	6 (6.6%)	7 (5.3%)	0	16 (4.3%)
Of those delivered	**Hull (n=96)**	**Leeds (n=85)**	**Sheffield (n=126)**	**Sunderland (n=53)**	**Total Delivered NAT-C (n=360)**
**Length of consultation (minutes)**					
Mean (SD)	25.7 (11.21)	24.5 (8.69)	26.5 (7.22)	17.5 (6.59)	24.4 (9.18)
Median	20.0	20.0	27.5	17.0	24.0
(Range)	(10.0, 60.0)	(16.0, 40.0)	(8.0, 60.0)	(8.0, 35.0)	(8.0, 60.0)
IQR	(20.0, 30.0)	(20.0, 32.0)	(22.0, 30.0)	(12.0, 23.0)	(20.0, 30.0)
Missing	15	74	0	0	89

[1] Only showing participants randomised to the NAT-C group (i.e. ignoring the one protocol violation where a participant in the usual care group received a NAT-C session)

Table 2 NAT-C performance by clinicians

**Clinicians[1] (n=32)**	**Number of NAT-Cs performed**
Mean (SD)	11.25 (8.95)
Median (Range Min, Max)	9 (1, 32)
IQR	(4, 16.5)

[1] 53 clinicians were trained, 32 clinicians performed at least one NAT-C, and 21 didn't perform any NAT-Cs.

Table 3 NAT-C consultations resulting in referrals to external services

NAT-C consultations	n=360
NAT-C consultations resulting in external referral	50 (13.9%)
**Which specialties were referred to[1]**	**n=50**
Social worker	1 (2.2%)
Psychologist	7 (15.2%)
Specialist palliative care service	10 (21.7%)
Medical oncologist	1 (2.2%)
Other	10 (21.7%)
No speciality given	17 (37.0%)
Missing	4
**Assessment priority (of referred)**	
Semi-Urgent (2-7 days)	3 (6.7%)
Non-Urgent (next available)	42 (93.3%)
Missing	5

[1] There were 21 participants with missing information

[2] There were 25 'referrals required' in sections 2-4 but 50 actual referrals total

Table 4 Highest action taken per section of the NAT-C

	Q2: Patient Wellbeing (n=360)	Q3: Carer (n=360)	Q4: Carer Wellbeing (n=360)	Overall (n=360)
None / Missing / N/A	117 (32.50%)	240 (66.67%)	272 (75.56%)	102 (28.33%)
Directly Managed	170 (47.22%)	108 (30.00%)	82 (22.78%)	179 (49.72%)
Managed by other care team	49 (13.61%)	9 (2.50%)	4 (1.11%)	54 (15.00%)
member	24 (6.67%)	3 (0.83%)	2 (0.56%)	25 (6.94%)
Referral Required				
