# Supplementary material for: Mixed methods process theory evaluation to explore the implementation issues of the Needs Assessment Tool-Cancer (NAT-C) in primary care for people with cancer
Source: BMJ Open. 2026 Apr 8;16(4):e113686. doi: 10.1136/bmjopen-2025-113686 (PMC13064150; doi:10.1136/bmjopen-2025-113686)
Supplement: online supplemental file 5 [file bmjopen-16-4-s005.pdf]

Supplementary file 5 Clinician and key stakeholders Interviews Themes and participant quotes

Main theme	Subtheme	Quotes
**1. The perceived value of the NAT-C *(Coherence)***	**i) Sensitive awkward questions/permission to ask and probe**	**C2** *"I think having a template allows you to do that a bit more easily, because you can say, you know, the idea of this session is to look for any other issues and we do want to look at the wider picture, and, you know, one of those things it says on the form is maybe about your finances, so I suppose it does provide a bit of an opener when actually it might seem a bit more random to just be like, oh, you know, tell me, are there any other issues, what about…?"*
		**C7** *"Yeah, I mean, it is definitely good to have the prompts 'cause, yeah, you would – you - you'd probably tend to focus on physical and psychological symptoms rather than any of the others. So yeah, it is helpful."*
		**C6** *"I was asking questions in areas that I wouldn't have done if I was just gone round to kind of visit them."*
		**S2 R3** *"It was easy to use and asked questions that would not usually be asked."*
	**ii) Beyond unmet need**	**C4** *" So I think from a personal level, it's - it's been really good understanding of – of how patients live with cancer and therefore probably improve my longer-term - my skills longer-term in doing that."*
		**C7** *" I think people like to talk about what's happening to them. A lot of people may have had issues that they want acknowledging, you know, could have perhaps been done better. So it's good to know, you know, for the future, for me. And yeah, I mean, I think they were quite able to talk about the fact that they're facing, you know, a shortened life and so, erm, yeah, it wasn't - it wasn't difficult. It was - it was nice to perhaps give them the time."*
		**C2** *" I know a lot of the focus of it was, can I sort this now or can I refer this, or does it need palliative care? Or, you know, and actually moving things forward. So I do agree with you, you know, it's trying to get things out into the open earlier, to then save it becoming a problem down the line totally, erm."*

	**iii) Other diagnosis and long-term conditions**	**C1** *"I think there's a, I think there's a broader area. I know it's a cancer tool, but I think there's a lot – there's a lot – You'd implement it in all directions, if that makes sense, and you could implement in palliative care for bronchiectasis and heart failure and things like that which would be really useful, too, as well"*<br><br>**K4** *"I think that, erm, I think that we are increasingly recognising cancer as a long-term condition, erm, for people, and I think we would incorporate it as one of the many long-term condition reviews that we do. Erm, and so I think practice nurses are really, really key in that. Erm, yeah. And district nursing teams for people who are housebound. I think that that sort of approach resonates with their general approach and what they're used to doing for other chronic diseases."*
	**iv) over other tools fits with consultation style skills**	**K5** "*The fact – the fact that it is kind of consultation skills-based, I think, is – is really, erm, it probably will speak to GPs very much."*<br><br>**C3** *"I've used these tools sort of on and off over the years, and sort of partly for the reasons that I've given, I-I really, I don't really like them. Erm, I feel they are quite constraining, so I completely buy into the idea of a needs assessment. And I like the idea of a tool associated with – with training to highlight areas of good practice. I must say, when templates pop up on, for example, SystmOne, I'm afraid I'm not really a rule-follower, I tend to look for ways to subvert them."*
	**v) Across patient stages of cancer journey**	**C1** *"It's the last six months of somebody's life where, you know, the patient's – they're really starting to decline, they can't – they can't work as much, they have lots and lots and lots of worries about the future, in terms of how will they cope, how will the family cope, and then that impacts on the family in terms of them coping, cos they can see this loved one in front of them starting to decline. So, I think it's more, I think, it'd be a lot more use in palliative care stage rather than necessarily the earlier in the diagnosis."*<br><br>**C1** *"Actually, it is a really helpful tool to have, CANAssess study. Er, you know, you can dip in, you can dip out of it, I suppose. Erm, I think it's useful for any – at any stage of cancer, but I think particularly more towards the end palliative care stages, really. I imagine that would be more helpful, because you can ask people a bit more receptive to asking – to you asking difficult questions when they really know…"*<br><br>**C2** *"Yeah, I mean, a few of mine that I spoke to were just sort of six monthly follow up with oncology or a*

		yearly check-up or something, so you're right, you know. I don't like to think of them sort of being neglected in some sense, but yeah, I think using the tool more perhaps for the newly diagnosed, the change in diagnosis, the end of life, maybe would pick up more, more things, sort of, you know, concerns, really."<br><br>**C4** *"definitely felt that, erm, early in the cancer journey would be a good time to do it, 'cos of many patients I spoke to said, 'Well, if you'd asked me this two years ago, I've had all these different problems that needed help and sorting out'.."*<br><br>**C5** "*..but it could be an argument for doing it later on, you know, later on in their – in their cancer journey, rather than earlier…Because – because actually that's when the unmet needs will arise, and I think, like I say, when people kind of – then that's when they feel a bit maybe forgotten sometimes, because the – the buzz dies down from secondary care, but we don't necessarily know that they've been handed over to us, because there – there's no real communication, so that's where they kind of get a bit more lost."*<br><br>**C5** *"a lot of that initial input dies down, but they're still generally under the care of the oncologist or whatever, and they still might be going in for, like, chemo every three months, you know, six weeks, erm, but just that initial care and attention has disappeared a little bit, because the Macmillan team will be busy with new diagnoses and, erm, they – they – they don't need as many scans and things so, yeah, I think that's where maybe there isn't the handover that needs to happen, because everyone still thinks they're under secondary care and being dealt with, but we don't know that they're just sat at home worrying about things, and that the – that the initial kind of fuss has died down. So maybe there's a – maybe that – that's where the NAT-C would come in nicely is, you know, secondary care saying, well, our Macmillan nurses are maybe taking a bit of a step back now, this patient currently is fine, but we recommend it might be a good idea for the GP to – You know, that this might be a point where the GP might want to check in, and – and maybe the NAT-C could be part of that consultation."*<br><br>**C6** *"I think, at diagnosis, if people were unwell and had a significant amount of symptoms, then that would be useful. So if they've been getting progressively worse for six months, presented late, had lost weight, got a cough, erm, were – maybe their mobility had gone down, their social contact gone down, and then walked up in A&E with a, erm, an acute thing and was then found to have a widespread metastatic terminal cancer, then yeah, that would be useful to do at that point of diagnosis. And I think it would fit very well with a palliative*

		care situation and I – I suspect a lot of the questions would be applicable to anyone at end of life, whether it was due to cancer or not. So end – stage COPD, end – stage heart failure, old age and frailty, a lot of the questions – I can't think of the questions that wouldn't be applicable: pain, anxiety"<br><br>**K7** *"I think it — it seems that probably more relevant in the post—diagnosis, erm, period and if we're looking at primary care, then it's probably at this certain points through the journey when it might be more relevant such as, erm, after diagnosis or a certain period of time after diagnosis, when contact with secondary care reduces and patients are — are more sat in primary care rather than secondary care."*<br><br>**K3** "*my gut feeling, having done cancer care reviews over the years, is they are extremely valuable and they offer lots of questions for patients. And it means that they might come back again. You know, what you've got to remember in general practice is, is it's not just about that one consultation, it's about when they might re-present. Erm, afterwards…"*<br><br>**K4** ***"****People find it very difficult to argue with the patient. If you've got evidence and the patient voice, I think that that – I think that disarms a lot of the politics."*<br><br>**K5** *"…if we look at the trust across England or across the UK, uptake is very variable, and some of that will be down to individual choice of, erm, pa – individual service user choice, but also some of that will be down to hearts and minds of, you know, the clinicians that are meant to be intervening with the intervention, and, you know, how they – how beneficial they see it."*
	**vi) Information sharing with team**	**K8** *"as a practice, you're now a multidisciplinary team led by a consultant in general practice, but with nurses, with physician associates, with healthcare assistants, duh-duh-duh. How could this tool help you as a team to share information, to share - develop a shared understanding of the priorities and concerns at the moment and - and to direct your patients, then on to extra - to - to additional help if they need it?"*<br><br>**K5** "*You know, I think there's definitely a shared decision-making element, which is certainly the core part of the PCN DES this – the personalised care element of the PCN DES this year, so shared decision-making is really important"*

	**vii) Training framework for cancer care**<br><br>**viii) Patients benefit supporting self-management**	**K5** *"So – I mean, it's educational, so it will speak – you know, it will – you know it will tick PDP and CPD and PDP boxes, which I think is good."*<br><br>**K5** *"I mean, the voice of the service user is more powerful than ever, X, so, erm, showing impact for individuals getting, you know, individual service users to speak out."*
**2. Champions are key *(Cognitive Participation*)**	**Enthusiasts/champions and Cancer Leads**	**C4 "..** *each practice will have a cancer lead GP, erm, and I think it's probably getting access to that cancer lead GP, erm, and giving them some direction on how to set it up in their practice*<br><br>**K3** *"I'm an advocate of EPaCCS, which is the End-of-life Palliative Care Co-ordination System, so we – we've – I've led with [name of GP] from [town] on that in our area, and other GPs like [name] in [region], so that we successfully launched that, probably the beginning of the pandemic. That – that caused a lot of uptake of extra EPaCCS across the system, which was really good, so… I'm also, erm, voluntary – a trustee at [hospice], erm, so involved with the board from that point of view, erm, and through my CCG role, worked closely with the Hospice and Hospice-at-home in [town], erm, and I suppose was involved in, erm, the single point of access that was set up through the pandemic and the increase of care in the community through the pandemic with [name of service] and the Macmillan nurses there."*<br><br>**K3** *"So, and a lot of work in general practice is based around the lead in that practice, and the interest within the practice to do something. Erm, and that can vary, and that can vary for lots of different reasons, purely because there might not be a GP with a particular interest, or it might be a smaller practice, erm, but with the advent of PCNs [primary care networks], I mean, I'm aware of some really good work in sort of, sort of [town] across to [town] with a cancer care coordinator and some of the stuff they've done over the last three years involving contacting the patient, offering a review, not just medical, but also benefits and all those type of things, has – has been quite pioneering in that area."*<br><br>**K3** *"So you almost then need a cancer lead within that PCN. So the way we're going is probably PCNs, but you need someone to champion it, too. So if I was to try to instigate this in practices across your kind of approach, my cancer leads within the practices, because I still think at the moment in [PCN], that's my way in."*<br><br>**K3** *" So it's a combination of someone championing it clinically, I would say; some admin support; and then*

		maybe a [name] or somebody like that that brings them into those appointments, if that makes sense, erm."<br><br>**K5** *"Erm, champions, for sure – so, identifying champions that might have, erm, influence locally as well. Erm, that's generally how – how we start these interventions really, you know, getting somebody on board who, you know, is a believer and has some local influence, so that, you know, erm, that might just be from individual interest, for this individual interest in personal care, individual interest in, erm, communication skills, individual interest in cancer probably ideally, or end-of-life."*<br><br>**K6** *"so our Project Co-ordinator, erm, trained a small group of volunteers to be able to, erm, deliver the holistic needs assessment, erm, and then as a result of that signpost them to, you know, create a sort of a care package for them."*
**3. Effectiveness evidence is important but influences implementation indirectly *(Cognitive Participation)***	**i) Evidence of clinical benefit hard to demonstrate**<br><br><br><br><br><br><br><br><br><br><br><br>**ii) Evidence influences NICE guidelines, which influence clinical practice but mainly through policy drivers**	**K5** *"Erm, so I'm not sure how important NICE would be. I mean, yeah, no, I think more having a tool with evidence that then trying to convince the people that make the decisions about what interventions we use in primary care is probably the route that would have most impact."*<br><br>**C6** *", people could potentially look at it and go, yeah, that's – there's some good questions there, I'll just incorporate them into what I do and, erm, change their routine practice by pinching bits a bit, but not necessarily filling in the template and therefore not being provable, which would still improve patient care, but not in a way anyone could actually, erm, kind of prove – which, you know, it still had benefit."*<br><br>**C8** *" if you've got the evidence there, then people are far - people can see the benefit and are – and are converted much easily and are willing to put the effort in, because they can see the patient benefit and the impact from that."*<br><br>**C5** *"Yeah, I think if it was in the NICE guidelines, it would – it would – or any – any kind of national guideline that would raise its profile and make it something that, you know, GPs recognise as being something that is established and kind of endorsed by the NHS, or even – even at an ICS level, maybe a local kind of guideline is another way of doing it, where it's, you know, say – say, for example, the Cancer Alliance might encourage and resource practices to take it on."*

		**C8** *"If it was NICE-recommended in terms of the Cancer Care Review and 'this is our advice with regards to that', and it was on sort of a CKS summary, highlighting the - the sort of cost-effectiveness and the - the benefit of u- to utilising the NAT-C, I think that - that would go a long way in terms of establishing that we know."*<br><br>**K4** *'It's the right thing to do for the patient', erm, but yeah, I think we have to be careful with cost-effectiveness arguments because it – depending on our audience, I suppose. If we want to talk to commissioners, they're all for it, but I think, erm, I think for the individual practitioners, they need something more to buy into."*
	**iii) value (personal experience of patient benefit) trumps effectiveness evidence in terms of individual clinician implementation**	**K4** *"it would be more about how it helps patients, from my personal perspective. You know, I think cost-effectiveness and things, if that is for the NHS as a whole, that may not necessarily be resonating with individual kind of provi – I know we're supposed to be working in a lovely integrated way at the moment, but we're not, and, erm, I have been into practices before to talk about care -planning for palliative care, and I have said to them, you know, 'This is the right thing to do. We will be saving money for the system', and then I kid you not, the medical director of a big federation of practices said to me, 'Right, K4, if I'm paid apples and the hospital are paid pears, you tell me why I should spend my apples to save their pears.'"*<br><br>**K4** *"My experience of working with CCGs [commissioners], it takes their clinical lead to go in and say, 'Look, this is the evidence base, this is why you need to commission with this'."*<br><br>**K7** *"However, even NICE — there's very few things that NICE mandate to be – to be used as assessment tools in anything, really. So even if it does come to us, it will be – it will be, erm, I think the right word here — it would be very good to use it, but not necessarily mandated..."*<br><br>**K5** *"Erm, so, real clarity of evidence of impact for sure. You know, whatever that is, even if you don't get health economic impact, real clarity of bullet-point evidence as to why there's benefits and patient-level benefits, workforce benefits and system benefits"*

**4. Adequate resources are fundamental for implementation beyond practice level** ***(Collective Action)***	**i) capacity of primary care workforce in the current context:**<br><br><br><br><br><br><br><br><br><br><br><br><br><br><br><br><br><br><br><br>**GP Staffing pressures**	**C3** *"I know this isn't a forum for politics, but you know, the primary care system was working – and because the number of doctors and the percentage of the NHS budget given to primary care has dropped massively over the last 10-15 years, is now not working. It – it can't deliver, but that doesn't mean it couldn't deliver, if adequately resourced. Something like the NAT-C, for me, is optimally placed in a well-resourced primary care system, and, yeah, of course te – secondary and tertiary care sectors have a role, but I certainly don't think they should lead on this kind of work."*<br><br>**K1** *"Yeah, I think it's quite dangerous to do the one problem per consultation. It doesn't work, and it just - all it does is move the work. So, if you've got someone in front of you and they want to discuss five things, well, we haven't got the capacity to offer five appointments. We barely have the capacity to offer one, so just get them all done in one, if you can."*<br><br>**K1** *"It's really difficult, X (name). It's really difficult to - to get GPs to adopt new things. Erm, we're very stuck in our ways, erm, and it's really difficult to get GPs engaged, especially at the moment, because the workload is - It's awful! It's just awful! Oh my God, if I could retire tomorrow, I would, and I'm only 48."*<br><br>**K3** *"So the problem at the moment for general practice is the busyness and the continuity, , erm, and I I-I suppose, from my point of view, I was a full time partner, I went to half-time and then I'm now in one – one day a week salaried, and I realised that really most of my clinical day-to-day stuff is about firefighting."*<br><br>**K8** *"…any change, introduction, you know this very well, needs capacity to do it, doesn't it? And - and practices - on individual - individual practices and all practices at the moment are on their knees in terms of – of - of capacity to - to do anything differently. There - We – we - I've talked with my colleagues often, so I'm not saying anything that I haven't said with them about everybody feeling like they're rats on a rat wheel"*<br><br>**K5** *"So, you know, I think – I think there's a practicality versus ambition there. And I think generally practicality wins, especially when it comes to primary care."*<br><br>**C3** *"All – all effective and cost-effective health care systems across the world internationally rely on strong primary care, and primary care, in my view, is the best place to deliver the majority of health care, including needs assessment for people with – with cancer, and the thing which practically makes that impossible at the*

		moment is lack of resources. So just like a million other unmet needs within the British population, the issue is lack of capacity in primary care, so if I was the chair of the LMC and you came to me and say, 'Look, we've got this intervention which we know is effective and cost-effective', I would say, 'Well, how many GPs does it need to deliver it, and can you tell me when the commission and arrangements will be put in place to provide those GPs?' And in the current context, that would be, there aren't any, and that would mean it won't happen."
	**Lack of time to train**	**K6** *"So I think the main one is – is the one is the – the – the essential resource of time and workforce. Who's going to do it? Who's got the time to do it within a job plan? Erm, can it be a nonclinical person? Can it be, you know, somebody administrative that's brought in to bolster the community, erm, team workforce? Erm, and – and does it generate work for the clinical team or does it just support – does it support the patient in a way that maybe makes them need less input from primary care?"*<br><br>**C2** *"You know, it's quite a complex-looking form and you've got to read through it all and try and work out what's what, and that takes a bit of time. And in order to probably do it well, you probably need to invest a little bit in it, and, you know, maybe have some training and maybe understand this and that, and, you know, we get training – 'Do this, do this', you know – for lots of different things, so it would be a priority amongst many others."*<br><br>**C3** *"so I think training would be important but, to go back to my issue with resources again, train- so often announced that training is available without any, erm, time to undertake that training, and then that itself becomes a bit box-ticky, because the training is not taken up, and therefore it doesn't achieve its aims."*<br><br>**K7** *"I think it would need quite a lot of structured training, erm, for a number of people within – within — within practices, so it's not just the clinician, it's often the — the back — back team knowing how that would fit in with their computer systems, erm, if it's a digital — a digital tool or there's elements of using digital, erm, options for it. So there's also the, erm, the infrastructure that goes with that that the back office needs to be aware of, but from a — an issuing point of view"*
	**Lack of time to deliver**	**C8** *"I'm sure they'll be able to see the patient benefit and some potential there, but then they will struggle with regards, how do we fit the same time etc., ll this cost implications of a long appointment. So there'll be - there'll - I'm sure there'll be significant pushback. So there'll – we'll really need a strong case to evidence the*

		benefit."
	**Individual capacity**	**K1** *"Erm, and I - Of course, all GPs would want to do this well, but, you know, we've got busier and busier and busier, erm, and are just firefighting all the time, so trying to get GPs to, you know, spend time doing a holistic, non-rushed, face-to-face cancer care review is almost impossible."*<br><br>**K7** *"So most consultation at GP surgery last about 10 to 12 minutes and it's whether something like this would actually be feasible within that time period or whether this needs a dedicated appointment; whether it needs dedicated time prior to the appointment, and how do you logistically arrange that and factor that in, and whether that in itself is feasible, erm, and — and that fits in with – There's so much pressure on appointments, is it — is it — it feasible? Is it cost—effective or time—effective to have additional appointments in order to do this prior to a meeting with the clinician, or do they need more than one appointment in order to do this? And is that an effective use of time within primary care, when we're already, you know, stretched to get appointments?"*
	**Nihilism don't want to find things can't fix and don't go looking**	**C7** *"Yeah. What do I do about it? Yeah, that's it. So where would I refer you on? You know what -? 'Cause it is all about referrals, isn't it, or whether they're self-managed? Yeah. So sometimes I think, well, I don't know if I want to - you know, you don't know if you want to try and sort of almost promise something and then say, well, there isn't anything I can do about that - Yeah, you know, type of thing."*<br><br>**K6** *"… Other top tips… I suppose people just being, erm, being aware of what – what it is and the limitations of it, because I think sometimes you can create a false promise, like, you're asking people all these questions, but you maybe aren't necessarily able to solve all the problems, and it's difficult to do that in a realistic way, but without being kind of defea–defeatist. But you don't want to raise false hopes, do you?"*
	**ii) financial resources, e.g., QOF, ,**	**C5** *"there probably would need to just be some funding up front to say, 'we realise this is going to take a bit of your management team – IT teams – time to set up, a bit of GP time to do a bit of training and get your head around it so we, you know, that there is a – a fixed amount of funding per practice that signs up for you to implement it, and, you know, get up and running with it'."*<br><br>**K5** *"but it's a challenge when there isn't – You know, unless you're coming in with a chunk of funding attached*

		to it, which we are used to now in primary care, even for quality improvement initiatives, we're used to, even if it's – Sometimes it's nominal funding, you know, it's not like anything that's really going to impact on the income of the practice, but there's just a recognition that this is over and above for, you know, for want of a better phrase."<br><br>**C4** *"Erm, Any of these things need to be, erm, associated with a payment, I think. That – and – and a points-based system. I think, really – I think GP practices are set up to – to respond to that kind of contract, erm, and you have… so I think that's the only way you'll get GPs to do it…, I think we all want to provide really good care to people. We don't want to box-tick. We – we don't want to just do things for the sake of doing them."*<br><br>**C6** *" So if it became the approved thing for QOF and you had to fill it in, then people would do, at least to the minimum degree that, erm, fitted with the QOF criteria."*<br><br>**C3** *"I see QOF as a way of paying GP practices for the extra intensity of admin and clinical work associated with delivering a service which aims to improve outcomes, you know, so I I like QOF and, erm, QOF comes through as a manual, you know, a handbook. It's very specific about what should happen, how it should happen, how often it could happen, what the associated remuneration is for it happening, erm, so I like that"*<br><br>**C5** *"Is – is this going to be something where there is a financial benefit to the practice of making sure they do it, or is it something that you would provide to GPs and say, 'This is – this is really useful, it's been demonstrated to be effective, we recommend you use it'."*<br><br>**C8** *"And I know all the sort of - all the - all the QOF things, and if it's in QOF, or if there's a payment linked to ticking the box or having completed, the GP seem to respond really well to that."*<br><br>**K1** *"Oh, definitely try and roll it out across primary care and I would definitely try and encourage the use of it by, you know, dangling carrots and using sticks, but the problem is that there – there aren't really any sticks you can use, erm, so you would just have to dangle carrots, erm, and it's how – it's how receptive is primary care is at the moment, erm, given the current state of affairs"*<br><br>**K3** *"And then you could, I mean, adding to that in terms of QOF and payments that people get for QOF and*

		cancer reviews, that always motivates to a degree, cos that causes income, erm, but it's not the be-all and end-all, if that makes sense."<br><br>**K8** *"But I can see it - I can see it having a place in - in ticking the QOF box."*
	**Danger of tick box exercise and poor completion of NAT-C**	**C3** *"What often happens is people pay lip-service to badly implemented – well, inadequately resourced – interventions and there's always wriggle-room to tick the box, erm, but not actually deliver what was envisaged, erm."*<br><br>**K3** *"we're starting to realise that QOF isn't the be-all and end-all, but QOF did serve a purpose. It did bring standards up in things like diabetes and that, things like this, whereas with cancer review, a lot of it is about holistic, opening up that patient, giving them more wellbeing advice, making them feel more comfortable and able to come back to ask, cos that's what you're trying to do."*<br><br>**C2**: *"I suppose the more templates and tick-box-y things that people get a bit twitchy about, don't they, and, you know, you kind of have to do this, is a bit more than just, erm, 'this might be useful if you think it would benefit your patients' sort of thing. I think it's just unfamiliarity, really, often. You know, people are nervous about using a new tool."*<br><br>**C4** "*We don't want to box-tick. We – we don't want to just do things for the sake of doing them"*<br><br>**C8** *"Yeah, subconsciously you feel yourself rushing and trying to get through it and then it becomes more of a tick-box exercise."*<br><br>**K1** *"You can't say, 'You must use this template'. You – you can't. There's no way of doing that or holding someone to account over it, though, say, QOF – cancer care reviews are in QOF…. but then there is no way of ensuring the quality behind that. It's a numbers game."*<br><br>**K3** *" it's about the busyness of primary care at the moment, and it's about how different practices do and perform and value their cancer care reviews, I suppose, because historically it's always been – Erm, I mean from a QOF point of view, they all get done, but they're all done to different degrees within different*

		practices."<br><br>**K4** *"Erm, so yeah, so for me, the tools are all very well, but actually the philosophy of care in the practice, the practitioners around the person, how that tool is used, that's where we need to be really, really focusing our efforts."*<br><br>**K4** *"And the fact in general practice that we're all, you know, I don't know - the culture is so much tick-box these days. We've slightly lost the plot with it, to be honest."*
	**Directory of services to refer to e.g. community, charity links**	**C3** *"….I sometimes find it difficult because I think the services that that the patients I interviewed needed either didn't exist or were difficult for me to sign post to. I'm thinking particularly about, erm, erm, I suppose, spiritual issues, dealing with your own mortality, erm, existential crisis linking with anxiety, depression; that to make any meaningful inroads on those kinds of issues I think would require, erm, a discrete counselling service, you know, with people with skills and expertise and, most importantly, time, you know"*<br><br>**C8** *" I think there'd be a lot of clinicians that would be uncomfortable about those discussions, and some training about, actually, if you pick up this need, there's these services or this support available to her, because you don't have to have the answers, you just need to know how, where to direct people and how to support them. So I think that it would be really useful having that as well. That would address some ser- some serious concerns."*<br><br>**K2** *" as far as things like knowing where services, what services are available, cos part of the problem is actually, how do we all know what's available, including, you know, the health service and practice nurses and all the rest of it? You only know what you know."*
	**iii) potential use of existing resources: role of PCNs could reduce workload on individual practices – network level, national**	**K3** *"I like the model in, erm, [PCN]] – which is, what, PCN, which is north of the [PCN], where they have a cancer coordinator who, when there's a diagnosis, they then contact the patient. Her name's [name]. She's brilliant. She – she speaks to the patient. She's not clinical. Erm, she asks them what their concerns are, asks them if they'd like a clinician appointment and goes through other things, gives them handouts if they need it, can facilitate CAB[citizen's advice bureau] and other things like that, and I-I think that is a logical role, and I know some of the PCNs are looking at similar roles, erm, and it's then about who you get them in to see."*

	**level.**	**K4** *"Erm, I think if you were to get a group of really bought-in professionals in something like a cancer alliance, then yes, there would be scope, and if, even better, if the – if the bought-in individuals were across all the different sectors, if there were primary care reps and secondary care people with a commitment to work together to make it work, then yes, I think that would have an impact locally."*
	**Use of current systems infrastructure already existing in community setting, charities; triage and**	**K5** *"that infrastructure really on the whole, should already be in existence and we would – you know, we would ideally try to fit in with that infrastructure so that we're not starting from scratch, trying to develop a community – you know, directory of volunteer organi – you know, especially and with ICSs now, you know, we've got voluntary service, erm, sector leads that should be linking in with all the voluntary sector services in that organisation, so there should be these robust networks and directors of services in place"*<br><br>**C5** *"I think GPs are usually the people who do the cancer care reviews, really, although it could be used by any – You know, it could be used by care co-ordinators or community nurses, advanced care practitioners, you know, anyone who's involved in the care of the patient. You could actually do the – you could actually have a NAT-C that is done by a care co-ordinator and then, you know, they have the option to refer to the GP if – if needed. You know, I don't know whether that's something that you would think about?"*
	**Use skill mix e.g., nurses, advanced clinical practitioners, non-medical cancer care co-ordinator, link worker;**	**K3 "***Yeah, potentially, yeah. I mean, obviously – I mean, practices have – I mean, the obvious – obvious parallels are COPD/asthma and diabetes. Erm, the number of patients they've got on – on their books are huge, so – so most practices will have a diabetes nurse or a CPD/asthma nurse who will see the majority of those patients, erm, and I think that's where a role would be useful to develop within that, erm, but depending on the size of the practice, it may be one or two nurses, erm, in terms of availability. Erm, you'd have to buy in the GPs as well, because I think some of the GPs will do that face-to-face consultation with them, but sometimes that can take on other – other modes."*

**5. NAT-C practicalities *(Reflexive Monitoring)***	**i) Training adequate, informative and remembered**	**C1** *"Yeah. Yep. That patient consultation video was good, actually. I watched I think it was a GP in X (place), wasn't it, who was doing the consultation? It was good, yeah."*<br><br>**C8** *"Yeah, I think the training was useful. It was interesting just to go through it because it's some - it's a tool I've not used before, wasn't aware of, and actually just be familiarising myself with it, going through it and – and had a discussion on the approach to use it and actually to use it as a tool to support discussion and allow the patient sort of to be able to express their needs utilising those questions, I think, was useful going through that in terms of the training and - and the advice on the sort of consultation style, conversational etc. I think was useful."*<br><br>**C8** *"I think I remember through the training I thought, oh, this is going to be really useful in terms of structural - structuring my consultation and asking those questions I would never ask in reality."*<br><br>**C2** *"Yeah, it's good to see in practice, certainly. Um, I do remember it's a home visit, wasn't it? And it was a patient and a wife, their husband or somebody, caregiver? Erm, yeah, no, I think it was fine, it's good. It's a practical example, isn't it, certainly? Erm, and having the form in front of us when we were then watching them, to think about what we would ask and how it relates to the template, is useful."*<br><br>**C2** *"Yeah, it was OK. I had originally thought that we were going to sort of block out separate, you know, an extra session where you just did all of these, whereas in the end they ended up being mixed in amongst all my other normal patients. So, you know, I've certainly perhaps didn't have as much time as I might have liked to have recapped the form and, you know, prepared myself a bit more, but that the lady who I did it with it was lovely and, you know, it was fine. It was nice. It was nice chat, you know. Uh, yeah, I think maybe that was probably more of a personal thing in terms of fitting it into my workload, not a lack of training, perhaps."*<br><br>**C4** *"No, I do remember the video, so it did stick in my mind, and I think those kind of - visual kind of aids are often good, aren't they? And – and - and so I do - I do remember, and it was - I think that was really useful seeing a GP deliver it in the way GP would deliver it. Erm, I thought that was, I thought that was effective."*
	**ii) Learning curve**	**C1** *"No, I think that it took me a little bit time to get my head around what I had to do, because I'm – what I am doing, I don't know whether rightly or wrongly, erm, I'm using the tool, but I'm just putting it, putting all*

		my information on a sheet of paper, and I'm just trying to transcribe it into what, which box I think that they fit, and I'm asking the patients, 'Are there any questions you have?' and, or 'Which bit do you think you fit into?' I find it a little bit clunky, but then that's just part and parcel of the way that it's made up, but I'm appending quite a big long spiel in the little box for additional notes, a bit of it."<br><br>**C7** "*Very clunky the first time, and then it becomes better as time goes on. So I - I wouldn't be too critical of the training. I think they did point that out, but you know, you sort of want to do a good job, you don't want to miss things, and you want to make sure you're covering things. So you – you know, yeah, you've gone exploring a bit too much."*
	**iii) IT Systems integration, visibility**	**C1** *"Yeah, yeah. I think it makes sense because it's just us having to – I don't – I always have – Currently, I always have to think about where is the CANAssess template? Where do I fill it in? Then I have to go down through six ? and look through different boxes to find it, so it's a bit – a bit tucked away at the minute."*<br><br>**C8** "*So we'd - we'd need some guidance on where you - where we're wanting to utilise it. We'd need the IT set up in terms of - Because at the moment, even though it's - it's straightforward to find it, but it does need you to go in and search for it. So it need linking in with whatever system the GPs are using. I know we have like a doctor's bag that Ardens gives us and then it's all on there, so it would need incorporating into established systems that we're using at the moment, whether that's Ardens or any other system, and, I think"*<br><br>**C4** *"we couldn't work out how to edit them once they're saved. Erm, that's – that's – that's an IT issue, whether we, yeah… if you saved the notes, you couldn't then go back into the notes, open the template that you'd done before and edit it. You had to open it in a new template and put some new information in, which was – There may be a way around that, but we couldn't work out how to do that."*<br><br>**C5** "*I think it's essential, actually. I think that if you – I think any tool these days, if you – if it's not integrated with the clinical system, the jobbing GP is just – isn't going to use it."*
	**iv) NAT-C template modifications,**	**C4** "*Yeah. OK. I – I wonder whether on the – on the actual template itself there's a kind of a text box at the bottom where you can write free text, but I was finding that I was writing free text for each different question, whereas if there was a free text bit for each question, then you could free text in the right place. Because*

	**simplification and embed in current systems**	*there's a few times where I was trying to label my answers to Question One, so I put 'One' and then I write something, then I put 'Two' and write something, and I might go and get confused between the questions, or just the way it's set up on the screen, having a text box for each question might be quite helpful, I thought."*<br><br>**C6** *"Erm, yeah, I think the issue with the templates is that it's tick – boxes are hard to capture subtlety, and patients are all complicated in kind of unique, erm, and a 'pain/no pain' box is – is fine, but it's a bit – it's very binary and you probably want to write something like, you know, 'gets some pain sometimes in the morning', a little bit of this, you know, use a bit of Oramorph every now and again, that works, no side effects, can manage, doesn't want more analgesia, which is easier to type than to try and find a load of boxes that can accurately reflect that."*<br><br>**C7** *"So if that was less busy, and I think, erm, the - the all the - all the information about all the questions about the carers and the family, I think, perhaps is a bit over - you know, not – not - It's a bit too much."*<br><br>**C8** ***"****Erm, I think what we – what - how you can work with templates is if there's a link for NAT-C on the - on the template, what people don't do is they won't open up one template and then open up another. But what they will do is if there's a link on there, there's a button on there to open up the NAT-C, then they're more likely to utilise it, and there's a lot of links or buttons on Ardens that open up other sort of scoring mechanisms, or sort of questionnaires, so if that was done, I think it - people were more likely to use it."*
