# Supplementary material for: Mixed methods process theory evaluation to explore the implementation issues of the Needs Assessment Tool-Cancer (NAT-C) in primary care for people with cancer
Source: BMJ Open. 2026 Apr 8;16(4):e113686. doi: 10.1136/bmjopen-2025-113686 (PMC13064150; doi:10.1136/bmjopen-2025-113686)
Supplement: Supplementary file 6 [file bmjopen-2025-113686.reviewer_comments.pdf]

PEER REVIEW HISTORY

BMJ Open publishes all reviews undertaken for accepted manuscripts. Reviewers are asked to complete a checklist review form and are provided with free text boxes to elaborate on their assessment. These free text comments are reproduced below.

ARTICLE DETAILS

Title (Provisional)

A mixed methods process theory evaluation to explore the implementation issues of the Needs Assessment Tool-Cancer (NAT-C) in primary care for people with cancer.

Authors

Swan, Flavia; Clark, Joseph; Blenkinsopp, John; Farrin, Amanda J; Wright-Hughes, Alexandra; McNaught, Emma; McCormack, Terry; Johnson, Miriam

---

VERSION 1 - REVIEW

**Reviewer**	**1**
**Name**	**Liu , Raymond**
**Affiliation**	**The Permanente Medical Group Inc**
**Date**	**14-Dec-2025**
**COI**	**None**

Thank you for the privilege of reviewing this manuscript.

The authors report using a mixed-methods evaluation of the NAT-C intervention in the CANAssess2 trial. Clinicians had to receive training and have conducted at least 2 NAT-C consultations to be included in the evaluation. Using paired surveys (before and after training / implementation) and qualitative analysis the authors note that the perception of NAT-C was generally positive to NAT-C before the training. After implementation providers found that it was difficult to integrate NAT-C into existing work and there was a need for functional IT systems for implementation and resources to address the identified needs. The authors do note that they were unable to recruit to their target interview sample and were not able to recruit any nurses as a major limitation of the work.

There is little known about the benefits of NAT-C and this manuscript addresses some important knowledge gaps. The following considerations could strengthen the manuscript.

Major comments:

- I would recommend shortening the manuscript and supplemental materials (in total 152 pages) to improve readability. Specifically:

o Is it necessary to include the entire CANAssess Protocol in the supplementary materials? It seems like it would be sufficient to have included the protocol as part of the main analysis on clinical effectiveness (reference 10).

o Line 96-99: It seems like a description of the results of the CANAssess The description of results could be more concise and moved to the background session rather than including it in methods. You should still include reference 10 but the framing in methods would be to mention that the details of the trial itself has been reported separately (reference 10).

o Box 1 would probably be adequate in the supplementary material rather than the main manuscript as the specific questions within the framework are repeated in Table 3 and further described after Table 3.

o The authors already include all the major quotes by theme in supplemental file 4 and then re-report many of them in the manuscript lines 253-243. Perhaps cut down on some of them in the main text to include at most 1-2 quotes for each major concept (right now it's mostly 2-3).

o It isn't clear how Figure 2 helps further explain the implementation issues – is it really needed?

Minor comments:

- Line 87: To make it clearer that this manuscript addresses the process evaluation I would recommend replacing "A process evaluation…" to something more specific to this manuscript such as "We undertook a process evaluation to explore…" or "This manuscript describes a process evaluation which explored…"

- Line 79: How does reference 4 support the claim for benefit and fidelity as the reference is related to a palliative care and cardiac topic?

- Line 192: Table 1:

o Do you have a distribution of median NAT-C per clinician and range to get a sense of how many each person performed?

o I don't think there needs to be a separate section of 99% live facilitated and 1% pre-recorded. This could adequately be noted in the table legend as "99% trained online" rather than a separate data section in the table.

o What is the significance of days between training and first NAT-C appointment - the data was reported in Table 1 but it wasn't clear how to interpret it and there was no mention of the data in the manuscript itself to help the reader understand why it was included. If it isn't important or difficult to interpret what the data mean, I wouldn't include it.

- Supplementary file 3 / Table 3: The formatting is off where the Referral required information doesn't match with any data.

- There was mention of a discrepancy between perception of % of unmet needs of the providers and the actual % of unmet needs reported by patients. This seems like a critical discrepancy – can you briefly discuss why you think that's this happened in the discussion?

**Reviewer**	**2**
**Name**	**Dixit, Niharika**
**Affiliation**	**UCSF, Medicine**
**Date**	**07-Jan-2026**
**COI**	**None**

I read the manuscript with great interest. Here are some of my suggestions.

1. On page 20, the quote from K8 is described as a training framework. While I agree that the quote describes a way to share information and whole-person care.I am not sure it is described as a training framework.

2. Theme no 3 does not seem to capture the complexity of data, you may consider rewording it ; I am not sure if this theme captures the complexity of the quotes. You may consider rewording it to make it more effective evidence while important but has a limited impact on implementation.

3. Theme 4, I think the first paragraph and quote may belong to the prior theme, as it talks about guidelines and not necessarily resources.

VERSION 1 - AUTHOR RESPONSE

Reviewer 1

Author's reply

We thank the reviewer for their positive review and detailed comments to shorten the manuscript. Please find below the revisions which have been made to address each comment individually.

Reviewer 1 Major Comments

There is little known about the benefits of NAT-C and this manuscript addresses some important knowledge gaps. The following considerations could strengthen the manuscript.
I would recommend shortening the manuscript and supplemental materials (in total 152 pages) to improve readability.
Specifically:

o Is it necessary to include the entire CANAssess Protocol in the supplementary materials? It seems like it would be sufficient to have included the protocol as part of the main analysis on clinical effectiveness (reference 10).

Author's reply

Thank you for your suggestion. We have removed the protocol - although we had thought this was required as part of the initial submission. It is true most if it refers to the main trial, however, it is the only place where details of the NPT parallel study are presented (we will leave the final decision with the editors).

Reviewer 1 Major Comment 2

o Line 96-99: It seems like a description of the results of the CANAssess The description of results could be more concise and moved to the background session rather than including it in methods. You should still include reference 10 but the framing in methods would be to mention that the details of the trial itself has been reported separately (reference 10).

Author's reply

Yes, we have moved the description of the published trial results to the introduction and have framed that the trial has been separately reported in the methods.

Reviewer 1 Major Comment 3

o Box 1 would probably be adequate in the supplementary material rather than the main manuscript as the specific questions within the framework are repeated in Table 3 and further described after Table 3.

Author's reply

Thank you for this suggestion we agree and have moved Box 1 to the Supplementary material file 3.

Reviewer 1 Comment 4

o The authors already include all the major quotes by theme in supplemental file 4 and then re-report many of them in the manuscript lines 253-243. Perhaps cut down on some of them in the main text to include at most 1-2 quotes for each major concept (right now it's mostly 2-3).

Author's reply

Thank you for drawing our attention to the duplication of quotes and your suggestions to reduce word count. We have limited the quotes in the main text to those that are needed to support the text. We have checked and removed all duplications in Supplementary file 5.

Reviewer 1 Comment 5:

o It isn't clear how Figure 2 helps further explain the implementation issues – is it really needed?

Author's reply

Thank you for your advice We had included it as a final schematic representation of the combined clinician and stakeholder interview findings, but as this is already described in text we are happy to delete as suggested.

Reviewer 1 Minor Comments

Comment 1- *Line 87: To make it clearer that this manuscript addresses the process evaluation I would recommend replacing "A process evaluation…" to something more specific to this manuscript such as "We undertook a process evaluation to explore…" or "This manuscript describes a process evaluation which explored…"*

Author's reply

Thank you for your suggestion. We agree that the wording recommended is clearer and have amended accordingly.

Comment 2

- Line 79: How does reference 4 support the claim for benefit and fidelity as the reference is related to a palliative care and cardiac topic?

Author's reply

We have checked reference 4 and believe that this is appropriately cited as the scoping review reports the results of what evidence is available for cancer care reviews (Gopal et al What is

the evidence behind cancer care reviews, a primary care cancer support tool? A scoping review. Published in J Cancer Survivors) – perhaps the reviewer misread?

Comment 3

- Line 192: Table 1:

o Do you have a distribution of median NAT-C per clinician and range to get a sense of how many each person performed?

Author's reply

Thank you for your data query. We have added text to the results to describe the summary statistics for the number of NAT-Cs performed per clinician as follows:

> "The NAT-C was performed at least once by 32/53 (60%) of the trained clinicians. The number of NAT-Cs performed by clinicians was mean (SD) 11.25 (8.95), median (min, max) 9 (1, 32), IQR 4,16.5). See Supplementary file 4, table 2."

and we have included the table below in Supplementary File 4.

Clinicians (n=32)	Number of NAT-Cs performed
Mean (SD)	11.25 (8.95)
Median (Range Min, Max)	9 (1, 32)
IQR	(4, 16.5)

Of 53 clinicians who were trained, 32 clinicians performed at least one NAT-C, and 21 didn't perform any NAT-Cs.

Comment *4*

o I don't think there needs to be a separate section of 99% live facilitated and 1% pre-recorded. This could adequately be noted in the table legend as "99% trained online" rather than a separate data section in the table.

Author's reply

We particularly had on-line prerecorded training available for practitioners unable to make facilitated sessions, therefore we would like to keep this to be transparent about our reporting and use of training methods, i.e., this method was used, but only rarely.

Comment 5

o What is the significance of days between training and first NAT-C appointment - the data was reported in Table 1 but it wasn't clear how to interpret it and there was no mention of the data in the manuscript itself to help the reader understand why it was included. If it isn't important or difficult to interpret what the data mean, I wouldn't include it.

Author's reply

Thank you for drawing our attention to the lack of reporting of the training data. We have added the following text to report the summary results:

> "The number of days between training and first NAT-C appointment were mean (SD) 105.7 (47.93), Median (Range) 97.0 (46.0, 230.0)"

The training results were highlighted in Table 6 Synthesized findings:

> "The time from training to delivery of the first NAT-C appointment was over three months and some clinicians only recalled the video consultation in any detail. They deemed the video to be an effective training tool."

Therefore, we have added the interpretation to the synthesized findings that:

> "Despite some clinicians having considerable time between training and delivery of the first NATC this did not appear to adversely impact their ability to do so."

Comment 6

- Supplementary file 3 / Table 3: The formatting is off where the Referral required information doesn't match with any data.

Author's reply

Thank you for drawing our attention to Supplementary file 4 / Table 3. We have checked and revised the formatting and headings to improve the clarity of the data presentation as below:

Supplementary File 4 Table 3 NAT-C consultations resulting in referrals to external services

**NAT-C consultations**	**n=360**
**NAT-C consultations resulting in external referral**	50 (13.9%)
**Which specialties were referred to[1]**	**n=50**
Social worker	1 (2.2%)
Psychologist	7 (15.2%)
Specialist palliative care service	10 (21.7%)
Medical oncologist	1 (2.2%)
Other	10 (21.7%)
No speciality given	17 (37.0%)
Missing	4
**Assessment priority (of referred)**	
Semi-Urgent (2-7 days)	3 (6.7%)
Non-Urgent (next available)	42 (93.3%)
Missing	5

[1] There were 21 participants with missing information
[2] There were 25 'referrals required' in sections 2-4 but 50 actual referrals total

Comment 7

- There was mention of a discrepancy between perception of % of unmet needs of the providers and the actual % of unmet needs reported by patients. This seems like a critical discrepancy – can you briefly discuss why you think that's this happened in the discussion?

Author's reply

We thank the reviewer for the suggestion. We agree it is an important point. We have checked that we have drawn attention to this in the discussion, but we have expanded the point a little as follows:

> "The NAT-C assessment helped identify, and plan action for, unmet needs in nearly two-thirds of intervention practice participants, despite a perception by clinicians that most had few needs. This supports the use of a systematic approach to 'uncover' otherwise hidden issues; clinicians may not 'see' significant problems and the patient may not volunteer them without prompts.(19, 20)"

Reviewer 2 Comments to Author

I read the manuscript with great interest. Here are some of my suggestions.

Comment 1

1. On page 20, the quote from K8 is described as a training framework. While I agree that the quote describes a way to share information and whole-person care.I am not sure it is described as a training framework.

Authors reply

Thank you for your comment. We agree that the quote does not address the training framework therefore we have added an additional quote from K8 where they explicitly described the tool as a training framework.

> ***K8*** *"So - so this tool - almost all this, you know, the tool almost becomes the framework to have a - a training session that's about the - why - why tailored care, whole person care, that - that recognises these multiple elements is – is – is - is appropriate."*

Comment 2

2. Theme no 3 does not seem to capture the complexity of data, you may consider rewording it ; I am not sure if this theme captures the complexity of the quotes. You may consider rewording it to make it more effective evidence while important but has a limited impact on implementation.

Authors reply

We agree and have reworded theme 3 to reflect your comment:

> "Theme 3. Effectiveness evidence while important has a limited impact on implementation."

Comment 3

3. Theme 4, I think the first paragraph and quote may belong to the prior theme, as it talks about guidelines and not necessarily resources.

Author's reply

Thank you for your comment. We admit we were not completely sure where this best fitted. However, after team discussion we have placed this under resources because the QoF is a mechanism for payment-for-service and the clinicians' discourse mirrored this; the QoF payment is made is for a review consultation which should take place after diagnosis. No clinical guidance is given other than signposting to existing templates. We fell on balance we would prefer to keep it here as a reflection of this narrative.

VERSION 2 - REVIEW

**Reviewer**	**1**
**Name**	**Liu , Raymond**
**Affiliation**	**The Permanente Medical Group Inc**
**Date**	**08-Feb-2026**
**COI**	

The authors have addressed all my concerns with the manuscript.
